# Epilepsy-associated digenic variants affecting an actin/mitochondria/glutamate pathway promote seizure susceptibility

**DOI:** 10.1172/JCI198696

**Published:** 2026-07-16

**Authors:** Shenzhao Lu, Mengqi Ma, Shabab B. Hannan, Mingxi Deng, Hu Chen, Zhijian Yu, Lindsey D. Goodman, Haein Kim, Yun Zhao, Sandeep Kumar Dubey, Wen-Wen Lin, Xueyang Pan, Debdeep Dutta, Vishnu Anand Cuddapah, Jill A. Rosenfeld, Xi Luo, Zhandong Liu, Joshua M. Shulman, Hugo J. Bellen

**Affiliations:** 1 Department of Molecular and Human Genetics, Baylor College of Medicine, Houston, Texas, USA.; 2 Jan and Dan Duncan Neurological Research Institute, Texas Children’s Hospital, Houston, Texas, USA.; 3 Department of Neurology, Baylor College of Medicine, Houston, Texas, USA.; 4 Department of Pediatrics, Baylor College of Medicine, Houston, Texas, USA.; 5 Baylor Genetics Laboratories, Houston, Texas, USA.; 6 Department of Neuroscience, Baylor College of Medicine, Houston, Texas, USA.

**Keywords:** Development, Genetics, Neuroscience, Epilepsy, Genetic diseases, Neurodevelopment

## Abstract

Epilepsy affects approximately 50 million people worldwide, yet more than half of individuals with a presumed genetic cause still lack a molecular diagnosis despite the identification of over 1,000 monogenic epilepsy genes. This diagnostic gap is unlikely to be resolved by improved variant detection alone, suggesting that variants affecting the same biological pathway may combine to cause disease. By studying epilepsy-associated actin regulatory genes, we identified a conserved actin/mitochondria/glutamate (AMG) pathway. We demonstrate that reduced actin polymerization promoted DRP1-mediated mitochondrial fission, increased ROS levels, and enhanced glutamatergic transmission, leading to seizures. The glial innate immune pathway, a recently recognized contributor to epilepsy, is activated when the AMG pathway is affected. Reducing mitochondrial fission with the mitochondria division inhibitor (Mdivi-1), or suppressing ROS with *N*-acetyl-l-cysteine amide (NACA), significantly alleviated seizures. Importantly, digenic heterozygous loss-of-function variants in AMG pathway genes combined to cause seizures, and individuals with epilepsy of unknown etiology showed an increased burden of such variants when compared with the controls. Modeling patient-specific digenic combinations in *Drosophila* confirmed that many combinations promote seizure susceptibility. Together, these findings establish the AMG pathway as a mechanistic framework for identifying digenic etiologies in epilepsy and highlight potential therapeutic targets.

## Abstract 2

<strong data-end=”778” data-start=”622”>We identified a new biological pathway associated with epilepsy and showed that losing one copy of each of two genes in this pathway can cause seizures.</strong>

## Graphical abstract

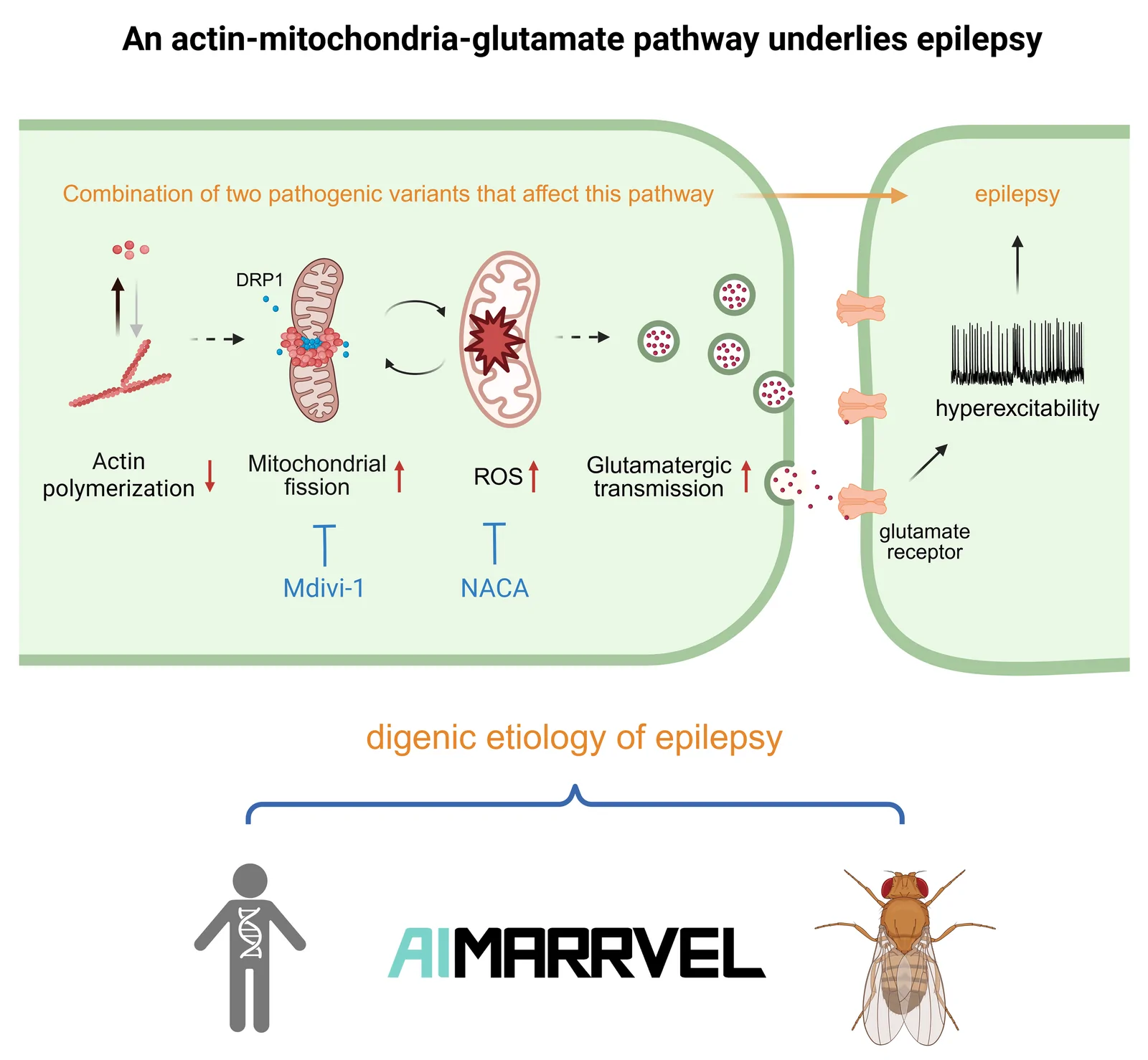

## Introduction

Epilepsy is one of the most prevalent neurological disorders, affecting 50 million people worldwide. Genetic studies have identified over 1,000 genes that cause monogenic epilepsies (1), enabling a diagnosis for 30%–50% of individuals with epilepsy (2–5). Hence, more than 50% of individuals with a presumed genetic cause of epilepsy are awaiting a genetic diagnosis (5, 6). This diagnostic gap is unlikely to be resolved solely by improving the detection and interpretation of variants on the basis of monogenic inheritance (7, 8). It is likely that combinations of some variants in specific genes play an important role in undiagnosed epilepsies. In addition, antiepileptic drugs are ineffective in more than 30% of individuals with epilepsy (9). This underscores the need to dissect the underlying molecular mechanisms to improve diagnostics and therapeutic interventions.

One of the main factors that limits our understanding of epilepsy at a mechanistic level is the involvement of numerous genes. Although ion channels and synaptic proteins that directly regulate neuronal activity are key contributors, the mechanisms underlying numerous epilepsy-associated genes remain poorly understood. Elucidating the molecular pathways responsible for these disorders and establishing the pathogenicity of variants in these genes will improve diagnosis and treatment for many unsolved and/or refractory cases.

Among the molecular pathways that remain poorly understood in epilepsy, actin dynamics have emerged as a potential contributor, and increasing evidence shows that variants in actin regulatory genes are associated with seizures (10, 11). Actin dynamics are essential for numerous cellular functions, including neuronal activity (10, 11). Actin dynamics involve the conversion of globular actin (G-actin) to filamentous actin (F-actin) or the reverse process, and it is tightly regulated by Rho GTPases. Plasma membrane receptors respond to extracellular signals to modulate the activity of Rho GTPases through the activation of Rho guanine nucleotide exchange factors (GEFs) (12). This GEF-GTPase activation promotes actin dynamics together with actin-binding proteins (12), which can either increase or reduce actin polymerization (13, 14).

An important GEF in the CNS is TIAM Rac1 associated GEF 1 (TIAM1). TIAM1 is highly enriched in neurons, where it regulates actin dynamics by activating Rac GTPase activity (15, 16). Notably, we recently showed that biallelic loss-of-function (LoF) variants in *TIAM1* cause developmental epilepsy (16). To investigate whether and how disruption of actin dynamics causes seizures, we utilized fly and human cell models for *TIAM1*-associated disease. We discovered an “actin/mitochondria/glutamate (AMG) pathway,” wherein actin polymerization defects lead to increased mitochondrial fission in glutamatergic neurons. This is associated with elevated ROS production. These defects lead to increased glutamatergic transmission and can be suppressed by inhibiting mitochondrial fission with mitochondrial division inhibitor 1 (Mdivi-1) or by reducing ROS with *N*-acetyl-l-cysteine amide (NACA). We also assessed whether other epilepsy-associated genes affecting this pathway may have similar effects. Interestingly, many transheterozygous mutant combinations of genes that affect the AMG pathway exhibit synergistic or additive effects leading to obvious seizures in flies, suggesting that digenic variants in the AMG pathway genes can combine to cause seizures. Furthermore, we found that individuals with epilepsy of unknown etiology had an increased frequency of the AMG pathway variants compared with controls. Modeling patient-specific digenic combinations in *Drosophila* confirmed that many combinations promoted seizure susceptibility. In summary, we identified an AMG pathway as a molecular mechanism underlying epilepsy that may improve digenic diagnostics and suggest therapeutic strategies.

## Results

### Behavioral defects in sif LoF mutants are mediated by glutamatergic and cholinergic neurons.

By querying the literature (1, 17) and the Online Mendelian Inheritance in Man (OMIM) database (18), we identified approximately 50 human genes that are involved in actin regulation and epilepsy among approximately 1,000 genes associated with epilepsy (Supplemental Figure 1A and Supplemental Table 1; supplemental material available online with this article; https://doi.org/10.1172/JCI198696DS1). Many of these correspond to downstream effectors of Rho GEFs and actin-binding proteins.

**Figure 1:**
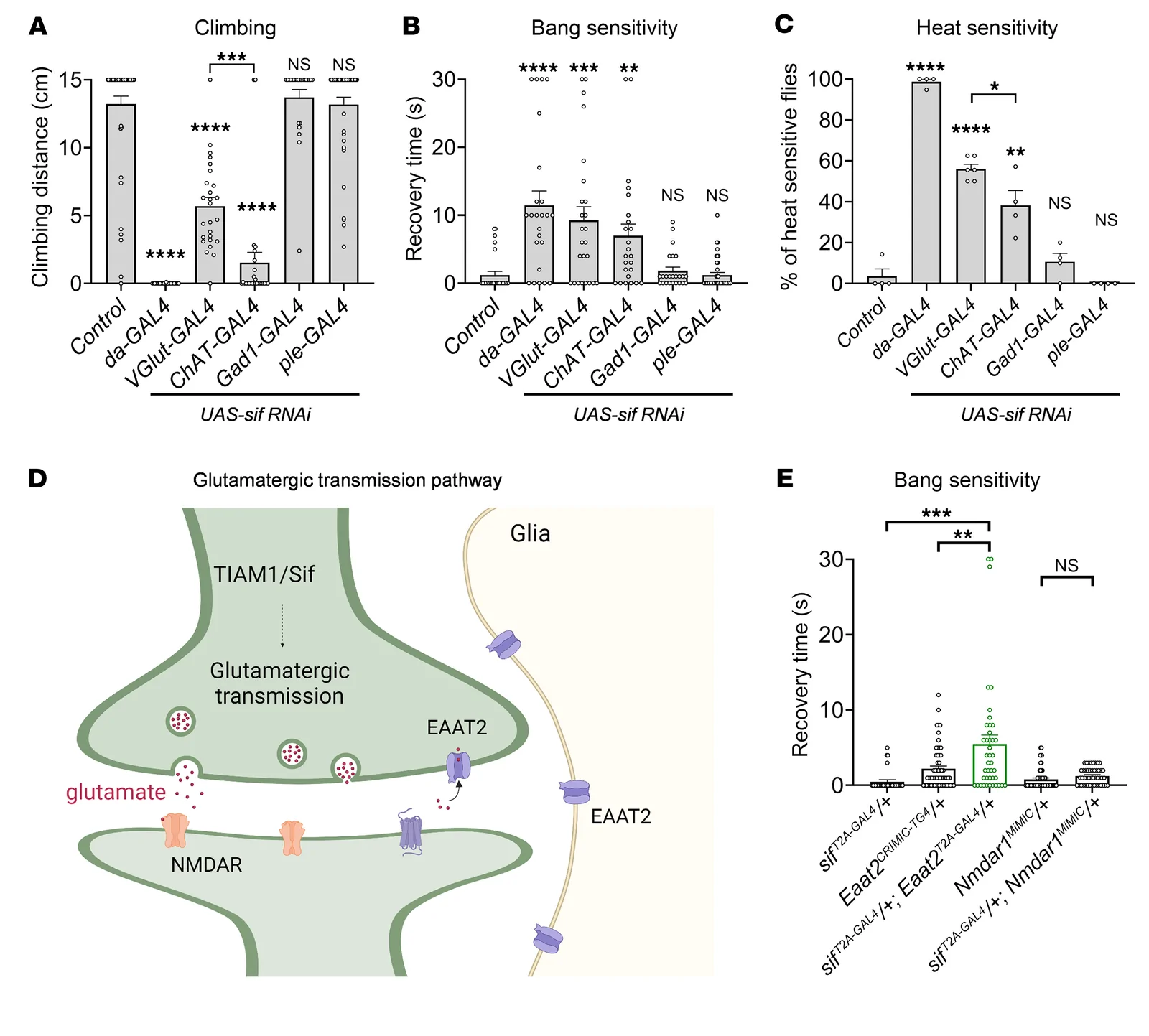
Behavioral defects in *sif* LoF mutants are mediated by glutamatergic and cholinergic neurons. (**A**–**C**) *sif* RNAi in glutamatergic neurons and cholinergic neurons led to climbing defects (**A**), bang sensitivity (**B**), and heat sensitivity (**C**) when compared with the control (*n* = 24–50 for **A** and **B**; *n* = 4–6 for **C**). Control: *da-GAL4>luciferase* RNAi. (**D**) Schematic of glutamatergic transmission (created with BioRender). NMDAR is a glutamate receptor. EAAT2 is a glutamate transporter that recycles glutamate from the synaptic cleft. (**E**) Genetic interactions of LoF alleles show that the transheterozygous combination of the *sif^T2A-GAL4^* allele with *Eaat2^T2A-GAL4^* allele causes a bang-sensitive phenotype (*n* = 32–59). Flies were tested at 3–5 days post eclosion (dpe). Data are presented as the mean ± SEM. *P < 0.05, **P < 0.01, ***P < 0.001, and ****P < 0.0001, by unpaired 2 tailed *t* test with Welch’s correction for unequal variances (**A**–**C**, and **E**).

Flies can be used to study the mechanisms underlying epilepsy. Mutations in fly orthologs of human genes that are associated with seizures often cause behavioral phenotypes consistent with human seizures, including mechanical stress–induced hyperactivation and paralysis (bang sensitivity) and/or heat stress–induced seizures and paralysis (heat sensitivity) (19–21). This hyperactivation of neurons has been confirmed, using electrophysiological studies, in some fly mutants that have seizures (22, 23). Furthermore, antiepileptic drugs used in humans often ameliorate these seizure-like phenotypes in flies (24–26). We previously identified individuals with variants in *TIAM1* who exhibited developmental delay and epilepsy (16) and showed that lack of *sif* (*still life*, the fly *TIAM1* ortholog) in neurons leads to seizure-like phenotypes, including bang and heat sensitivity (16). Since TIAM1 plays an important role in actin dynamics (15, 27) (Supplemental Figure 1B), we explored the phenotypes associated with loss of *sif* in fly neurons. We validated the notion that ubiquitous expression of a *UAS-sif* RNA interference (RNAi) (~88% knockdown efficiency) can induce severe phenotypes (Figure 1, A–C) that are very similar to those observed in *sif*-mutant animals (16). We next determined which neuronal cell populations require *sif* by selectively inducing expression of the *UAS-sif* RNAi in specific neuronal cell subtypes using established Galactose-responsive transcription factor (GAL4) drivers (Table 1). Interestingly, the *sif* RNAi in cholinergic neurons caused the most severe climbing defects (Figure 1A). However, *sif* RNAi in glutamatergic neurons caused the most severe bang sensitivity and heat sensitivity, but milder climbing defects when compared with the cholinergic *sif* RNAi (Figure 1, A–C). In contrast, knockdown of *sif* in either GABAergic neurons or dopaminergic neurons did not induce these phenotypes. These data indicate that *sif* was required in glutamatergic neurons and cholinergic neurons for proper neuronal activity. Given that glutamatergic and cholinergic transmissions can be excitatory in both humans and *Drosophila*, whereas GABAergic transmission is primarily inhibitory (28, 29), the above observations indicate that *sif* loss may lead to an increase in excitatory synaptic transmission.

Since glutamatergic *sif* RNAi causes the most severe seizure-like phenotypes, we further assessed the sensitivity of glutamatergic neurons to *sif* loss. We first considered whether loss of *sif* could genetically interact with genes required in the glutamatergic network — *Eaat2* and *Nmdar1* (Figure 1D). Loss of EAAT2, a glutamate transporter that is required for glutamate clearance, causes developmental epilepsy due to an accumulation of glutamate in the synaptic cleft (30). Interestingly, transheterozygous (*GeneA^LoF/+^ GeneB^LoF/+^*) flies carrying 1 copy of a strong LoF allele for *sif* and *Eaat2* exhibit more severe bang sensitivity when compared with the single heterozygous flies (Figure 1E, detailed genotypes are provided in Supplemental Table 2). Hence, *sif* loss enhanced the seizure phenotype caused by *Eaat2* loss, probably by promoting glutamatergic transmission. Loss of *Nmdar1*, which encodes a glutamate receptor, should decrease synaptic transmission (31) and hence may not interact with *sif*. Indeed, we found that transheterozygous flies with a single LoF allele of *Nmdar1* and *sif* did not exhibit bang sensitivity (Figure 1E). In summary, these data indicate that glutamatergic transmission is at the root of the bang sensitivity associated with *sif* loss.

### sif loss causes hyperexcitability of glutamatergic neurons.

To assess whether the activity of glutamatergic neurons is increased upon *sif* loss, we generated *VGlut-GAL4>CaLexA* flies. CaLexA induces a calcium-dependent nuclear import of LexA, which is stimulated by Ca^2+^-regulated dephosphorylation and nuclear translocation of nuclear factor of activated T cells (NFAT) (32). It includes 2 components: UAS-mLexA-VP16-NFAT and lexAop-rCD2-GFP. In the presence of VGlut-GAL4 and increased Ca^2+^ activities, GFP will be expressed and localized to the membrane of glutamatergic neurons. As a result, *sif* RNAi flies showed a significant increase in GFP intensity in the CNS, including the brain lobes and the ventral nerve cord (VNC), when compared with the control RNAi (Supplemental Figure 2, A and B). These data indicate that *sif* loss in glutamatergic neurons led to increased neuronal activity.

**Figure 2:**
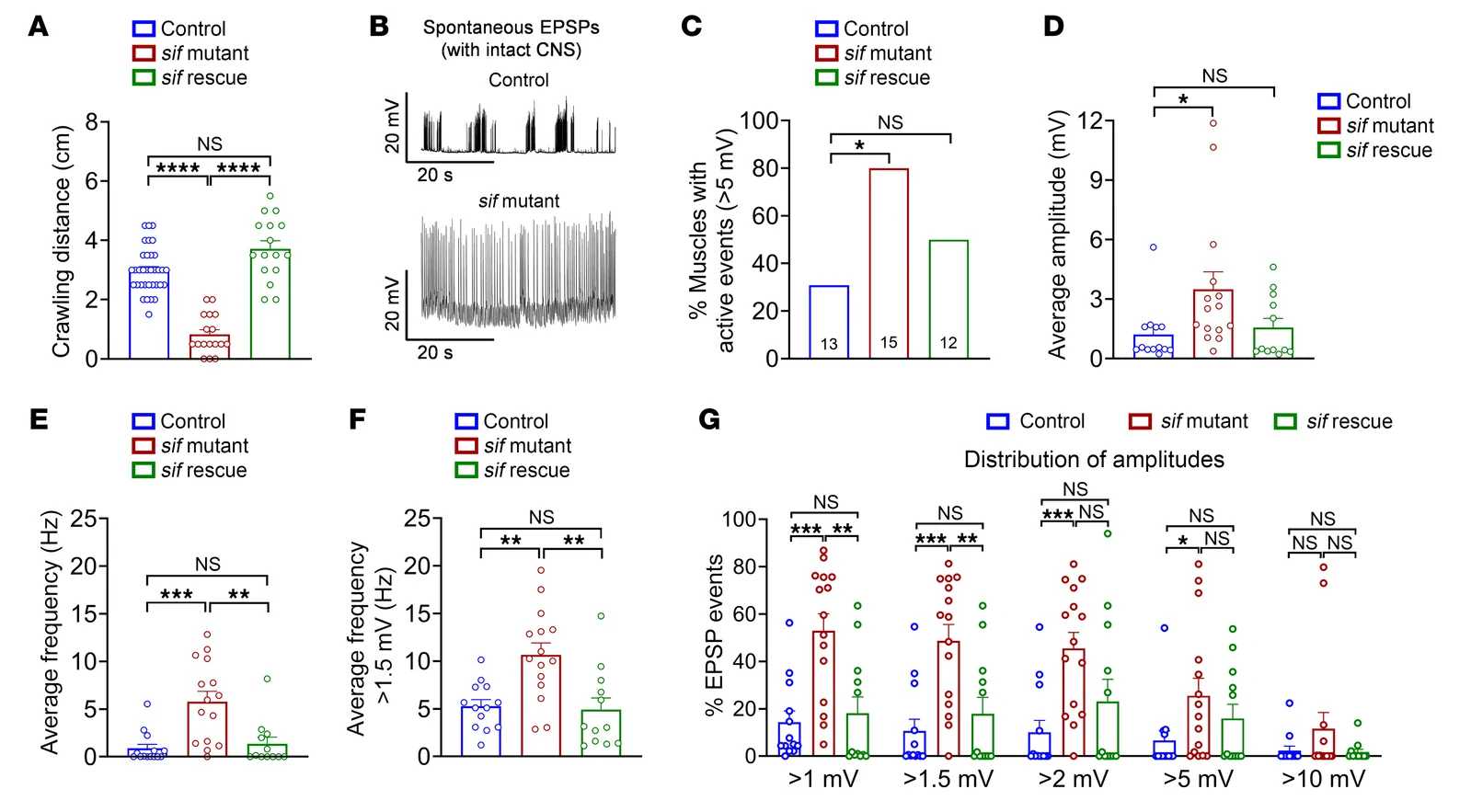
*sif* mutants display hyperexcitability at neuromuscular junctions. (**A**) The L3 larva crawling assay. Larvae were placed on an agar plate and allowed to crawl for 1 minute, and the crawling distance was measured (*n* = 15–34). (**B**) An electrode was placed on L3 larval muscles 6/7 in the abdominal segment, and spontaneous EPSPs were recorded for 2 minutes. Representative traces for spontaneous EPSPs with an intact CNS are shown. (**C**) The percentage of muscles with active events (>5 mV) were calculated. Fisher’s exact test. (**D**) Average amplitude of spontaneous EPSP events. (**E**) Average frequency of spontaneous EPSP events. (**F**) Percentage of events of greater than 1.5 mV for each muscle. (**G**) Distribution of EPSP amplitudes. Recordings were performed in 1 mM Ca^2+^ for 2 minutes. *n* = 12–15 cells recorded for each genotype. Genotypes: control (w1118), sif mutant (w; UAS-lacZ/+; sif^T2A-GAL4^/Df), sif rescue (w; UAS-sif/+; sif^T2A-GAL4^/Df). Data are presented as the mean ± SEM. *P < 0.05, **P < 0.01, ***P < 0.001, and ****P < 0.0001, by unpaired 2 tailed *t* test with Welch’s correction for unequal variances (**A**, and **D**–**G**).

Given that synaptic transmission at the fly neuromuscular junction (NMJ) is mediated by glutamate and an increase in synaptic transmission at larval NMJs has previously been shown to cause crawling defects (22, 23, 33), we evaluated crawling behavior of *sif*-mutant larvae. Control third instar larvae crawled approximately 3 cm in a minute (Figure 2A). In contrast, *sif*-mutant third instar larvae only crawled approximately 0.8 cm in a minute. The *sif^T2A-GAL4^* is a severe LoF allele (16) and produces GAL4 under the endogenous *sif* regulatory elements, which allows expression of the *UAS* cDNA in the proper spatial and temporal pattern of *sif* to assess the rescue effect (16). We observed that the crawling defects were fully rescued by expressing WT *sif* cDNA in the mutant background (Figure 2A, *sif* rescue), demonstrating that this phenotype was due to *sif* loss. These data suggest a defect in either muscle function, synaptic transmission, or both during larval development.

To determine if there are morphological defects at the larval NMJ, we stained the NMJs with HRP, a neuronal membrane marker (34), and *syt* EGFP, a synaptic vesicle marker (35). We did not observe obvious changes in fluorescence of HRP or *syt* EGFP when compared with controls (Supplemental Figure 3, A–E). Hence, *sif* did not obviously affect the neuronal morphology or distribution of synaptic vesicles in third instar larvae.

To assess the electrophysiological properties of NMJs, we performed intracellular recordings of third instar muscles. Consistent with a previous study (36), we did not observe obvious differences in the average amplitude and frequency of miniature excitatory postsynaptic potentials (mEPSPs) in *sif* mutants when the axons were severed (Supplemental Figure 4, A–C). We therefore recorded without severing the axons of glutamatergic motor neurons from the CNS. In control animals, approximately 30% of the muscles showed patterned EPSPs in a 2-minute recording period (Figure 2, B and C). These periodic contractions were induced by the CNS pattern generator, which drives muscle contractions (37, 38). In contrast, 80% of the *sif*-mutant larvae exhibited large, nonperiodic bursts of EPSPs in a 2-minute recording (Figure 2, B and C). Hence, the frequency and amplitude of EPSPs were significantly increased in *sif-*mutant muscles when compared with controls (Figure 2, D–G). Note that the increase in glutamatergic transmission was significantly rescued by expression of *sif* cDNA (Figure 2, D–G). In summary, our data indicate that loss of *sif* increased glutamatergic transmission without altering NMJ morphology.

### Sif loss promotes seizures by affecting actin polymerization.

**Figure 3:**
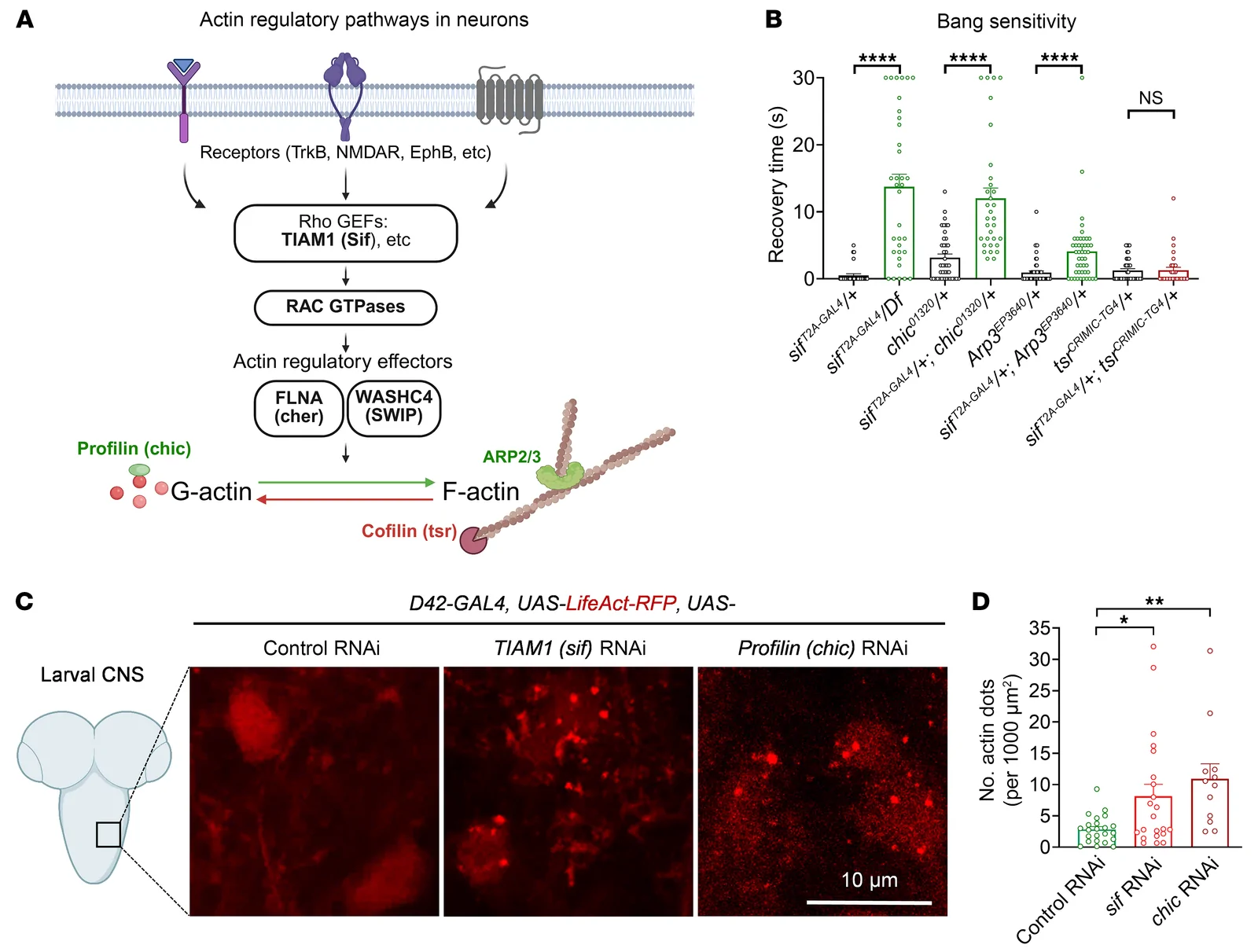
Actin polymerization defects are associated with seizures. (**A**) Graphical illustration of actin regulatory pathways in neurons (created with BioRender). Profilin (chic) promotes actin polymerization. The ARP2/3 complex nucleates actin filaments into branched networks. Cofilin (Tsr) mediates actin depolymerization. (**B**) Transheterozygous flies with combined LoF alleles of *sif* and *chic* displayed more severe bang sensitivity when compared with single-heterozygous flies (*n* = 32–56). (**C**) Glutamatergic motor neurons of the VNC from *sif* RNAi and *chic* RNAi animals. Actin filaments were visualized by LifeAct-RFP. Created with BioRender. Scale bar: 10 μm. (**D**) Quantification of the density of actin dots in neuronal cell body in the VNC (*n* = 12–23). Flies were tested at 3–5 dpe. Data are presented as the mean ± SEM. *P < 0.05, **P < 0.01, and ****P < 0.0001, by unpaired 2 tailed *t* test with Welch’s correction for unequal variances (**B** and **D**).

To determine whether the seizure-like phenotypes of *sif* mutants are due to a disruption in actin dynamics, we assessed dominant interactions between *sif* and other actin regulatory genes. As a GEF, TIAM1/Sif regulates actin remodeling by activating the Rho GTPase RAC1. The downstream effectors of Rho GTPases regulate actin filaments together with the actin-binding proteins that promote actin polymerization, including profilin (fly Chickadee, Chic) and actin related protein 2/3 complex (ARP2/3) (Figure 3A). We assessed *chic^01320^/+* (a LoF allele) flies and observed that they had mild bang sensitivity, whereas *Arp3^EP3640^/+* (a LoF allele) single-heterozygous flies did not show obvious bang sensitivity (Figure 3B). However, transheterozygous animals that carried a single LoF allele of *sif* and *chic* exhibited a severe bang-sensitive phenotype (Figure 3B). Similarly, *sif* and *Arp3* transheterozygous flies were also bang sensitive (Figure 3B). These phenotypes were also observed using another LoF of the *chic^221^* allele and the *Arp3^515FC^* allele (Supplemental Figure 5A). We also tested cofilin (fly Twinstar, Tsr), a protein that disassembles actin filaments (13) (Figure 3A). Neither *tsr^T2A-GAL4^/+* (a LoF allele) flies nor the *sif^T2A-GAL4^/+ tsr^T2A-GAL4^/+* transheterozygous flies displayed obvious bang sensitivity (Figure 3B). These data indicate that the loss of *sif* affected bang sensitivity by reducing actin polymerization and that other proteins required for proper actin polymerization also play a critical role in seizures.

We next aimed to visualize the defects in actin filaments in neurons using an established reporter, *Lifeact*-RFP (39, 40). Lifeact is a 17 amino acid peptide that binds to 2 consecutive actin subunits on the F-actin filament in cells and tissues (40), and its intensity is proportional to the amount and length of F-actin present (41). Knockdown of *sif* in motor neurons leads to an accumulation of small “actin patches” in cell bodies. These resemble actin patches in yeast (42) and correspond to short actin filaments. Interestingly, we also observed this phenotype when we knocked down *chic* (Figure 3, C and D). Since profilin (Chic) is known to promote actin polymerization, these actin patches likely reflect a reduction in actin polymerization levels. Hence, these data provide compelling evidence that loss of *sif* affects actin polymerization as previously shown in mammalian cells (27).

### Actin polymerization defects promote seizures in flies.

Numerous genes encoding proteins that regulate actin dynamics are associated with developmental epilepsy. GEF proteins regulate the actin cytoskeleton by activating Rho GTPases like *RAC1* and *RAC3*, and variants in these genes have been associated with seizures in humans (43, 44). In addition, although mice that lack *Rac1* or *Rac3* do not exhibit seizures, double-homozygous-KO mice exhibit spontaneous seizures (45). We therefore performed genetic interaction studies using 2 fly *Rac* alleles: *Rac1^J11^* (a LoF allele) and *Rac2^KG05681^* (a LoF allele), and 1 LoF *chic* allele. Both *Rac1^J11^* and *Rac2^KG05681^* synergized with *chic^01320^*, and the transheterozygous flies were bang sensitive (Supplemental Figure 5B, Supplemental Video 1). These data are consistent with seizures resulting from a disruption to a GEF/RAC GTPase pathway that reduces actin polymerization.

We next assessed whether loss of 1 copy of 2 different genes that promote actin polymerization can similarly affect seizure sensitivity. *WASHC4* (fly *SWIP*) encodes a subunit of the WASH complex that promotes actin polymerization (46), and *FLNA* (fly *cher*) encodes an actin cross-linking protein filamin A, which stabilizes polymerized actin filaments (Figure 3A). Pathogenic LoF variants in both *WASHC4* and *FLNA* have been associated with seizures in humans (1, 46, 47). Both *SWIP^CR70302^/+ chic^01320^*/+ and *cher^MiMIC^/+ chic^01320^*/+ flies exhibit increased bang sensitivity when compared with *chic^01320^*/+ flies (Supplemental Figure 5B and Supplemental Videos 2 and 3). In contrast, single-heterozygous loss of *SWIP* or *cher* alone does not cause obvious bang sensitivity. These data further indicate that disruptions in actin polymerization are associated with increased bang sensitivity and that digenic loss of genes regulating actin polymerization increases seizure susceptibility.

To determine if the actin regulatory proteins cause seizures by affecting glutamatergic transmission, similar to loss of *TIAM1/sif*, we performed genetic interaction studies with genes encoding proteins that are known to affect glutamate uptake at the fly NMJs. The previously tested mutations — *Rac1^J11^*, *Rac2^KG05681b^*, *SWIP^CR70302^*, and *cher^MIMIC^* alleles — all caused an obvious increase in bang sensitivity when combined with a single *Eaat2^T2A-GAL4^* LoF allele (Supplemental Figure 5C). Together, these data provide evidence that seizures can be caused by reduced actin polymerization levels in glutamatergic neurons.

### sif loss leads to an accumulation of small mitochondria in glutamatergic neurons.

Next, we asked how actin polymerization defects affect neuronal activity. Since actin dynamics are important for neuronal migration and axonal formation, we tested whether *sif* loss leads to neural wiring phenotypes. Our previous work has shown that *sif* is enriched in the antennal lobe during the developmental stage (16). We therefore examined whether the neural wiring of olfactory projection neurons (PNs) was affected (48, 49). As a result, loss of *sif* in the PNs did not cause obvious defects in neural wiring (Supplemental Figure 6, A–E). We also assessed the neural wiring pattern in the mushroom body using *OK107-GAL4*–driven GFP (50). Likewise, *sif* RNAi in the mushroom body also did not cause any significant changes in neural wiring (Supplemental Figure 6, F and G). These data suggest that *sif* loss may not obviously alter the neuronal projections and synapse formation during development.

**Figure 4:**
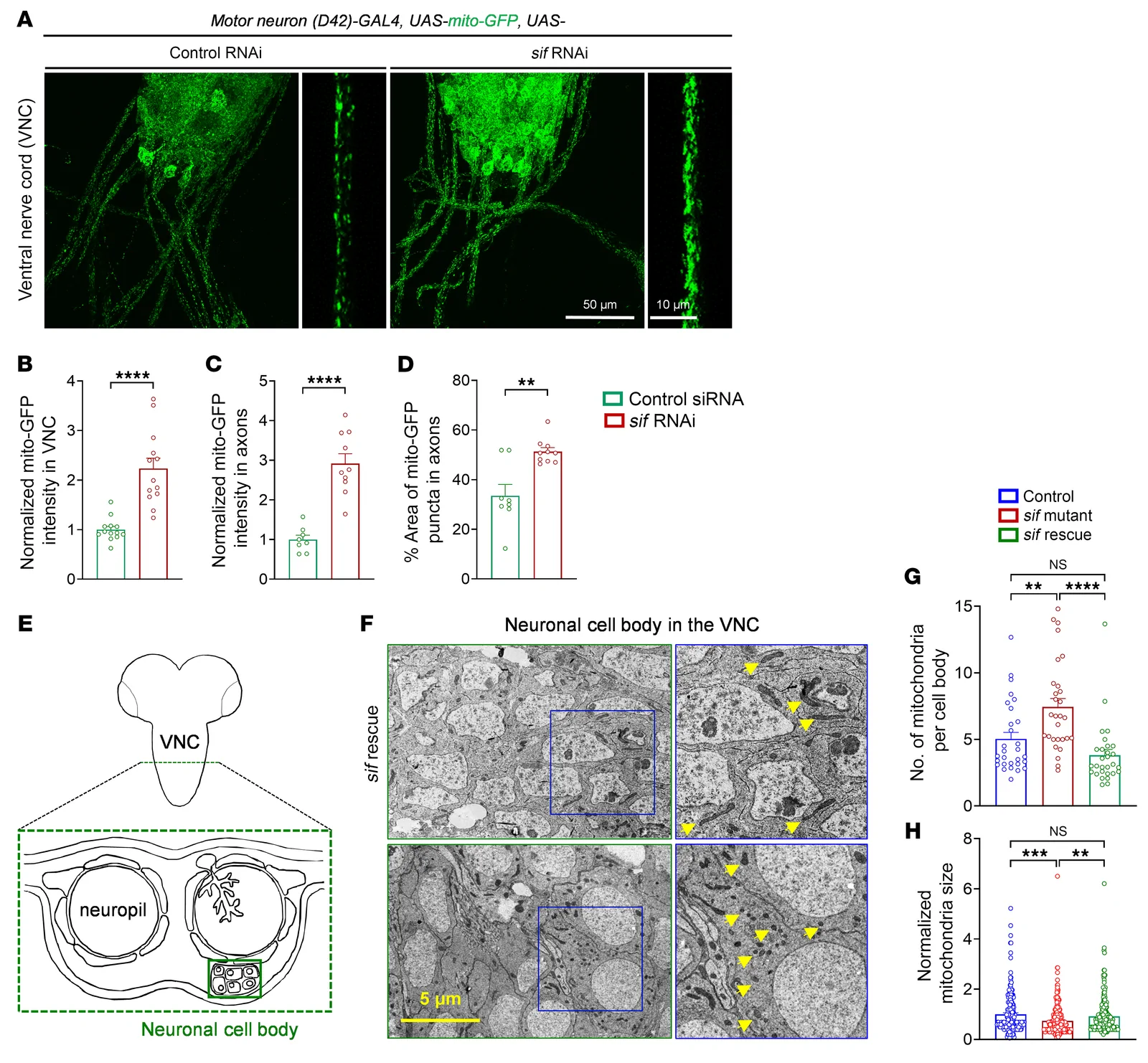
*sif* loss leads to an accumulation of mitochondria in glutamatergic neurons. (**A**) Representative images of third instar larval VNC cell bodies and axons of glutamatergic motor neurons from control (luciferase) RNAi and *sif* RNAi animals. Mitochondria are visualized by mito-GFP. (**B**) Quantification of normalized mito-GFP intensity in neuronal cell bodies (*n* = 13). Scale bars: 10 μm and 50 μm. (**C** and **D**) Quantification of normalized signal intensity (**C**) and percentage of area (**D**) of mito-GFP in neuronal axons (*n* = 8–10). (**E**) Diagram of the CNS of third instar larva. The CNS was dissected, and a transverse section was made for TEM. (**F**) Representative VNC neuronal cell body TEM images of *sif*-mutant and *sif* rescue neuronal cell bodies. Yellow arrowheads indicate mitochondria. Scale bar: 5 μm. Original magnification, ×1.85. (**G** and **H**) Quantification of mitochondria numbers (**G**) (*n* = 29 cells) and size (**H**) (*n* = 7–8 cells) in the VNC for WT, *sif* rescue, and *sif*-mutant animals (*n* = 3 animals for each genotype). Genotypes: *Canton S* (WT); *w* *UAS-sif/+ sif^T2A-GAL4^/Df* (*sif*-mutant); and *w* *UAS-lacZ/+ sif^T2A-GAL4^/Df* (*sif* rescue) Data are presented as the mean ± SEM. **P < 0.01, ***P < 0.001, and ****P < 0.0001, by unpaired 2 tailed *t* test with Welch’s correction for unequal variances (**B**–**D**, and **G** and **H**).

In addition to neuronal morphology and neural circuit regulation, the actin cytoskeleton participates in a variety of other cellular activities, including the regulation of mitochondrial function. Experimental paradigms have documented how cytoskeletal dynamics affect mitochondrial function by affecting their motility, fission, and mitophagy (51–55). We therefore assessed mitochondrial distribution using mito-GFP (56) in flies with *sif* loss. Interestingly, we observed an increase in the mito-GFP signal in both the soma and axons of glutamatergic motor neurons in *sif* RNAi when compared with the control RNAi (Figure 4, A–D). We also performed transmission electron microscopy (TEM) analysis of cross-sections of the VNC of *sif* mutants and observed a significant increase in the number and a decrease in the size of mitochondria when compared with the WT control (Figure 4, E–H). The mitochondrial phenotypes observed in *sif* mutants were rescued by expression of the *sif* cDNA (*sif* rescue) (Figure 4, E–H). These data indicate that *sif* loss perturbed normal mitochondrial dynamics.

Next, we assessed mitochondrial membrane potential using tetramethylrhodamine, methyl ester (TMRM) staining of *sif*-mutant brains and did not observe a significant change in TMRM intensity, indicating that these mitochondria maintained their membrane potential (Supplemental Figure 7, A and B). To further evaluate mitochondrial function, we measured ATP production. ATP levels in the adult fly head of *sif* mutants showed an approximately 1.5-fold increase when compared with levels in *sif* rescue flies (Supplemental Figure 7C). These data suggest that the mitochondrial function was not overtly compromised.

### Increased number and decreased size of mitochondria are associated with elevated Drp1 levels.

**Figure 5:**
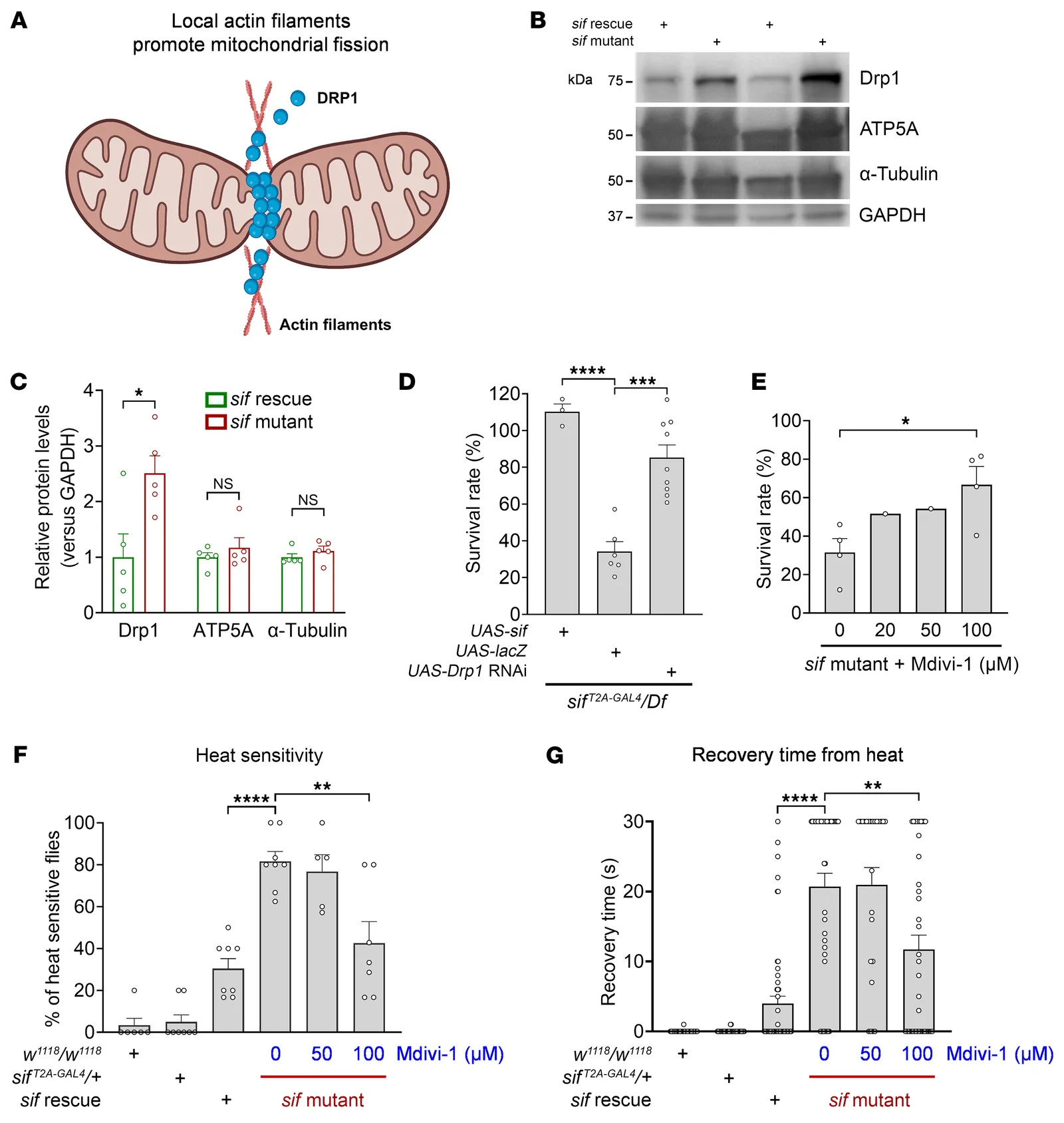
Inhibition of mitochondrial fission significantly rescues *sif* LoF phenotypes. (**A**) Graphical illustration of actin filaments mediated mitochondrial fission via recruitment of DRP1 (created with BioRender). (**B**) Protein levels in *sif* rescue and *sif*-mutant animals were determined by Western blotting. Third instar larvae were collected for protein extraction and analysis. (**C**) Quantification of protein levels. Data points represent the relative protein levels per independent repeat (*n* = 5). (**D**) Survival rate of *sif*-mutant animals with the expression of *UAS-Drp1* RNAi. The survival rate was measured using the expected Mendelian ratio by counting flies that eclosed and were viable after 24 hours. (*n* = 3–9, with over 300 progenies counted for each genotype). (**E**) Survival rate of *sif*-mutant animals fed different doses of Mdivi-1 (0, 20, 50, and 100 μM in food) (*n* = 4 for 0 μM and 100 μM groups, *n* = 1 for 20 μM and 50 μM groups). (**F** and **G**) Quantification of heat sensitivity for control (*w^1118^*), *sif* heterozygote (*sif^T2A-GAL4^/+*), *sif-*mutant (*w* *UAS-lacZ/+ sif^T2A-GAL4^/Df*), and *sif*-mutant flies fed different doses of Mdivi-1. The flies were tested at 3–5 dpe. Data points in **F** represent replicates (*n* = 5–8), and each replicate contains 5–10 animals; data points in **G** represent the recovery time for each animal (*n* = 24–54). Data are presented as the mean ± SEM. *P < 0.05, **P < 0.01, ***P < 0.001, and ****P < 0.0001, by unpaired 2 tailed *t* test with Welch’s correction for unequal variances (**C**–**G**).

During mitochondrial fission, the ER forms a ring around the fission site. Upon calcium release from the ER, G-actin is recruited to this site, where it forms short filaments. These short filaments recruit and bind the mitochondrial fission regulator dynamin-related protein 1 (DRP1), promoting its oligomerization and GTPase activity (51–55, 57) (Figure 5A). However, the relationship between actin dynamics, mitochondrial fission, and epilepsy has not been established.

Given that the mitochondrial number was increased but mitochondria size was decreased in flies lacking *sif*, we speculated that mitochondrial fission may be increased by increased Drp1 levels. We first assessed Drp1 protein levels and found that they were indeed upregulated in *sif* mutants (Figure 5, B and C). To assess whether this elevation contributes to the phenotypes, we reduced *Drp1* levels in *sif* mutants. *Drp1* RNAi significantly rescued the viability of *sif* mutants at 1 day post eclosion (dpe) from approximately 30% to 80% (Figure 5D), but it did not significantly improve the climbing defects or bang sensitivity (Supplemental Figure 8, A and B). However, we have previously documented that loss-of-function mutations in *DNM1L* (also known as *Drp1*) cause severe seizures in both humans (58, 59) and flies (60) (see Discussion). As actin polymerization is also required for targeting damaged mitochondria to the autophagosome (51, 61), we next considered whether other genes that affect mitophagy might play a role. We found that expression of *Pink1* or *parkin*, genes that promote mitophagy (62, 63), did not affect the survival rate of *sif* mutants (Supplemental Figure 9). These data argue that the increase in the number of mitochondria and the decrease in their size in *sif* mutants were, at least in part, associated with elevated mitochondrial fission driven by increased Drp1 levels.

Given that the *Drp1* RNAi improves *sif*-mutant survival but does not suppress seizures, we next assessed whether feeding *sif* mutants a small compound, mitochondrial division inhibitor 1 (Mdivi-1), was effective. Mdivi-1 decreases the GTPase activity of DRP1 without significantly reducing total DRP1 protein levels (64, 65). Here, Mdivi-1 treatment not only significantly improved the survival rate of *sif* mutants in a dose-dependent manner (Figure 5E) but also suppressed the heat sensitivity in surviving *sif-*mutant adults at the 100 μM dose (Figure 5, F and G). Together, these data indicate a pharmacological benefit of Mdivi-1 in our fly model for *TIAM1*-associated epilepsy.

### Elevated ROS causes seizure-like behaviors in flies.

**Figure 6:**
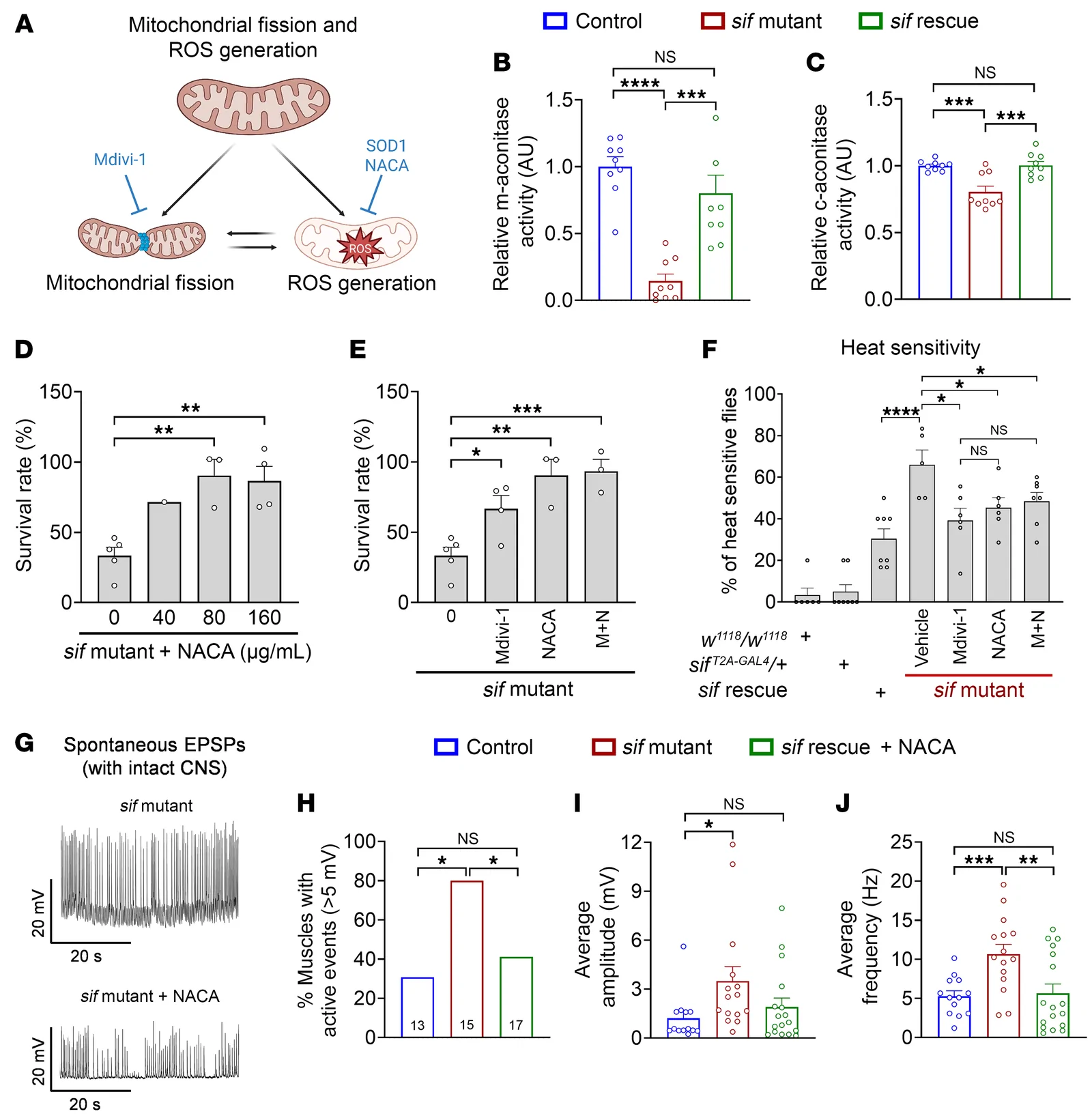
Inhibition of mitochondrial ROS using NACA significantly rescues *sif* LoF phenotypes. (**A**) Graphical illustration of mitochondrial fission and ROS generation (created with BioRender). (**B** and **C**) An aconitase activity assay was used to reveal high ROS in *sif* mutants. This high ROS was rescuable by expression of *sif* cDNA in these mutants (*n* = 9). (**D** and **E**) Survival rate of *sif* LoF mutants fed with different doses of NACA (0, 40, 80, and 160 μg/mL in food) (**D**) or Mdivi-1 and NACA (M+N) (Mdivi-1: 100 μM; NACA: 80 μg/mL) (**E**). *n* ≥3, except for NACA: 40 μg/mL group (*n* = 1), with over 200 progenies counted for each group. (**F**) Quantification of heat sensitivity for control (*w^1118^*), *sif* heterozygote (*sif^T2A-GAL4^/+*), *sif*-mutant (*w* *UAS-lacZ/+ sif^T2A-GAL4^/Df*), and *sif*-mutant flies fed Mdivi-1 (100 μM), NACA (80 μg/mL), or their combination (M+N). Flies were tested at 3–5 dpe. (**G**) Representative traces of spontaneous EPSPs from muscles with intact CNS of *sif* mutants fed normal food (top) or NACA (bottom). (**H**) Percentage of muscle cells with active events (events >5 mV). Fisher’s exact test. (**I**) Average amplitude of spontaneous EPSP events (*n* = 13–17). (**J**) Average frequency of spontaneous EPSP events (*n* = 13–17). Data are presented as the mean ± SEM. *P < 0.05, **P < 0.01, ***P < 0.001, and ****P < 0.0001, by unpaired 2 tailed *t* test with Welch’s correction for unequal variances (**B**–**F**, **I**, and **J**).

Since increased mitochondrial fission leads to elevated ROS (66, 67), and high ROS is associated with hyperexcitability and toxicity (23), we assessed if there is increased ROS production in *sif* mutants (Figure 6A). We measured the enzymatic activity of the citric acid cycle protein mitochondrial aconitase (m-aconitase). Both m-aconitase and cytosolic aconitase (c-aconitase) are highly sensitive to ROS-induced damage and have reduced activity in high ROS environments (68, 69). Indeed, the enzymatic activity of m-aconitase in *sif* mutants was decreased by approximately 86% when compared with control animals (Figure 6B), reflecting increased mitochondrial ROS. Furthermore, we observed that c-aconitase activity was also reduced by 20% (Figure 6C), indicating a mild increase in cytoplasmic ROS levels. Importantly, these reductions in aconitase activity were rescued by expressing a *sif* cDNA in the *sif*-mutant flies (Figure 6, B and C). To address whether ROS are elevated using another assay, we performed MitoSOX staining of fly brains. We found that *sif*-mutant brains displayed a significantly increased MitoSOX signal, indicating elevated ROS (Supplemental Figure 10, A and B). Finally, we used a third ROS indicator, *UAS-mito-roGFP2-Orp1* (70, 71), and tested for ROS in glutamatergic neurons and GABAergic neurons. *VGlut-GAL4>sif* RNAi flies exhibited an increase in mitochondrial oxidation in the brain (Supplemental Figure 10, C and D). However, *Gad1-GAL4>sif* RNAi flies did not show altered mitochondrial oxidation in the brain (Supplemental Figure 10, E and F), consistent with their lack of seizure phenotypes when *sif* was reduced in GABAergic neurons (Figure 1, C and D).

It has been reported that oxidative stress in neurons leads to upregulation of the glial innate immune response (IIR) genes, which in turn contributes to the progression of epilepsy (70). We therefore evaluated the expression of IIR genes. Consistent with previous findings, we observed that many IIR genes were significantly upregulated in the head of *sif* mutants when compared with the *sif* rescue group (Supplemental Figure 11). In summary, *sif* loss significantly increased the expression of IIR genes.

To further determine the effect of ROS on *sif* loss–induced seizures, we genetically increased ROS by reducing the levels of superoxide dismutase 1 (SOD1), an enzyme that breaks down superoxide radicals (72), using a *sod1^T2A-GAL4^* LoF allele. *sif^T2A-GAL4^/+ sod1^T2A-GAL4^/+* transheterozygous flies were more bang sensitive than *sod1^T2A-GAL4^*/+ flies (Supplemental Figure 12A). Together, these data argue that elevated ROS production indeed contributed to seizures in *sif* mutants.

To assess whether ROS may also underlie seizure phenotypes in other epilepsy-associated actin regulatory genes, we performed genetic interaction tests between the *sod1^T2A-GAL4^* allele and additional actin regulatory genes. Transheterozygous flies carrying the *sod1^T2A-GAL4^* allele and the *Rac1^J11^*, *Rac2^KG05681b^*, *SWIP^CR70302^*, or *cher^MiMIC^* alleles showed bang sensitivity (Supplemental Figure 12A). Given that these genes also dominantly interact with *Profilin* (*chic*) and *EAAT2* (*Eaat2*) (Supplemental Figure 12B), they seem to share a pathway defined by an AMG pathway in epilepsy.

### Inhibition of ROS by NACA significantly rescues the phenotypes of sif mutants.

We next determined whether the phenotypes of *sif* mutants can be ameliorated by pharmacological inhibition of ROS. NACA is a potent antioxidant that crosses the blood-brain barrier (BBB) and reduces ROS (73). We raised *sif* mutants on NACA-containing food or control food. We observed that NACA treatment significantly improved the survival rate of *sif* mutants in a dose-dependent manner, with 80 μg/mL NACA having the highest rescue ability: it improved the survival rate from approximately 30% to approximately 90% (Figure 6D). Combining Mdivi-1 and NACA did not further improve the survival rate (Figure 6E). We also tested the behavioral rescue and found that NACA treatment significantly ameliorated the heat sensitivity phenotype (Figure 6F). Mdivi-1 combined with NACA did not further improve the heat sensitivity phenotype (Figure 6F). These data argue that mitochondrial fission defects and ROS affect the same pathway.

To test whether NACA ameliorates seizures by reducing hyperexcitability, we performed NMJ electrophysiological recordings. The *sif* mutants were raised on NACA food (80 μg/mL), and the third instar larvae with an intact CNS were used for the recordings. Spontaneous EPSPs were significantly inhibited in the NACA-treated group, and the percentage of muscles with events above 5 mV were significantly decreased (Figure 6, G and H). The amplitude and frequency of EPSPs were significantly reduced in the NACA-treated group (Figure 6, I and J). Hence, reducing ROS via NACA significantly reduced glutamatergic transmission in *sif* mutants.

### Key features of the AMG pathway are observed in mammalian cells with TIAM1 siRNA.

**Figure 7:**
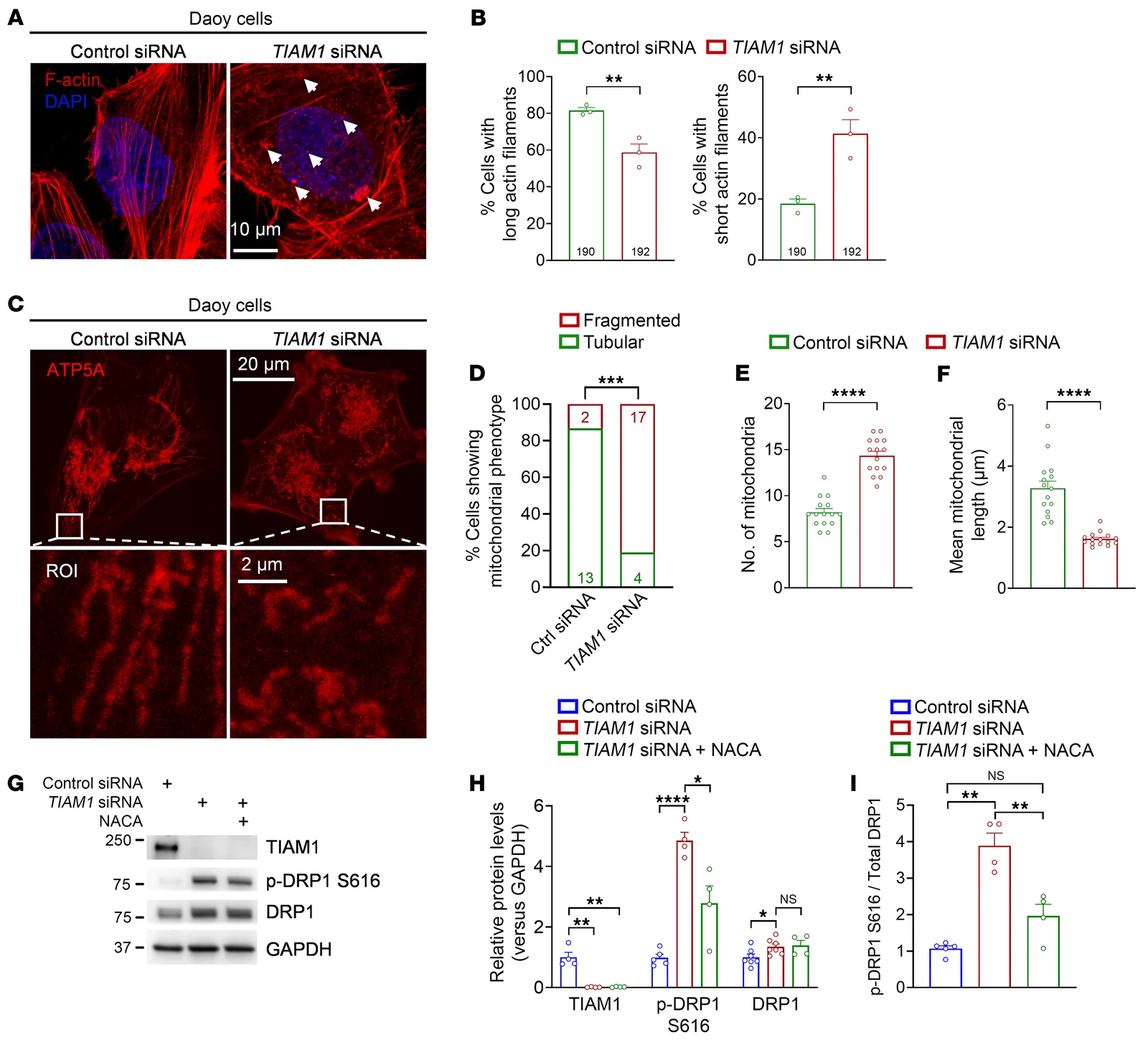
Knockdown of *TIAM1* in mammalian cells leads to reduced actin polymerization and increased mitochondrial fission. (**A**) Representative image of actin filaments in Daoy cells transfected with either a negative control siRNA or an siRNA targeting *TIAM1* (*TIAM1* siRNA). Red, phalloidin; blue, DAPI. (**B**) The percentage of cells with long and short actin filaments was quantified (*n* = 3 with 190–192 cells for each group). (**C**) Representative images of the ATP5A-labeled mitochondria in Daoy cells. (**D**) Quantitative analysis of the mitochondrial phenotype in each cell (*n* = 15–21). Fisher’s exact test. (**E** and **F**) Quantitative analysis of the number of mitochondria (**E**) and mean mitochondria length (**F**) in the region of interest (ROI) of each Daoy cell (*n* = 15). (**G**) Western blots of Daoy cells transfected with control siRNA or *TIAM1* siRNA and treated with or without NACA (750 μM, 24 hours). (**H**) Quantifications of protein levels indicate a significant increase in p-DRP1 (S616) and total DRP1 protein levels in the *TIAM1* siRNA group (*n* = 4–7). (**I**) The elevated p-DRP1 (S616)/total DRP1 ratio in *TIAM1* siRNA cells was significantly rescued by NACA treatment (*n* = 4). Data are presented as the mean ± SEM. *P < 0.05, **P < 0.01, ***P < 0.001, and ****P < 0.0001, by unpaired 2 tailed *t* test with Welch’s correction for unequal variances (**B**, **E**, **F**, **H**, and **I**).

To determine if the AMG pathway is conserved in mammals, we performed *TIAM1* RNAi in the human Daoy neuronal cell line (74) using a previously validated *TIAM1*-targeting siRNA (75). We found that this siRNA downregulated TIAM1 protein expression to less than 10% in Daoy cells (Supplemental Figure 13, A and B). Staining for F-actin using phalloidin revealed a significant decrease in long actin filaments and an increase in short actin filaments when compared with cells transfected with a control siRNA (Figure 7, A and B). We also assessed mitochondrial morphology using ATP5A immunostaining. *TIAM1* siRNA significantly increased the number and decreased the length of mitochondria in Daoy cells (Figure 7, C–F), consistent with what we observed in fly neurons. In addition, we also observed a significant increase in total DRP1 protein levels upon *TIAM1* knockdown (Figure 7, G and H), as well as a robust increase in phosphorylated DRP1 at Ser616 (p-DRP1 S616) protein levels (Figure 7, G–I). Note that DRP1 (S616) phosphorylation promotes mitochondrial fission (76). The elevation of p-DRP1 S616 was significantly rescued by treating the cells with NACA (Figure 7, G–I) or Mdivi-1 (Supplemental Figure 13, A–C). These data are consistent with our findings in flies, indicating a conserved AMG pathway in flies and humans. Overall, the data reveal an AMG pathway that may be disrupted in some individuals with seizures.

### Higher frequency of heterozygous LoF variants in AMG pathway genes in the seizure cohort.

**Figure 8:**
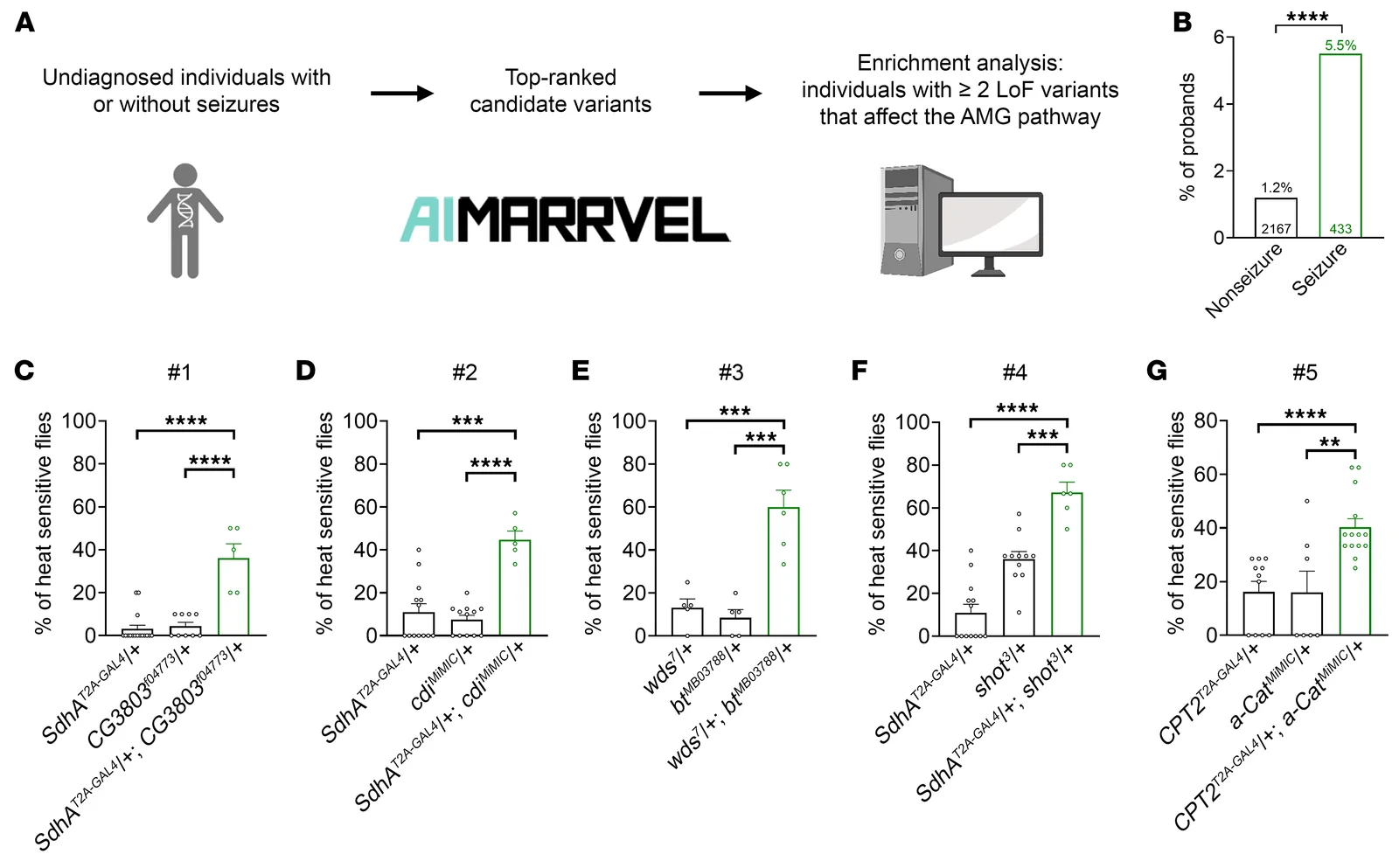
Increased burden of heterozygous LoF variants in AMG pathway genes among individuals with undiagnosed epilepsy. (**A**) Sequencing data of undiagnosed individuals with or without epilepsy were analyzed and the variants were ranked by AI-MARRVEL (v.1.1.0rc). The top 10 variants were used for the enrichment analysis. Created with BioRender. (**B**) The percentage of individuals with 2 or more LoF variants from the AMG gene set were counted. Fisher’s exact test. (**C**–**G**) Heat sensitivity of adult flies. The combinations of the LoF alleles show synergistic or additive effects for heat sensitivity (*n* = 5–14). Flies were tested at 6–9 dpe. Heat-sensitive flies are counted after heating at 42°C for 40 seconds (**C**), 50 seconds (**D**–**F**), or 60 seconds (**G**). Data are presented as the mean ± SEM. **P < 0.01, ***P < 0.001, and ****P < 0.0001, by unpaired 2 tailed *t* test with Welch’s correction for unequal variances (**C**–**G**).

Besides the approximately 50 actin regulatory genes associated with epilepsy, there are approximately 140 genes associated with epilepsy that are involved in the regulation of mitochondrial function, and approximately 30 genes that affect glutamate synthesis and transmission based on the literature (1, 17, 18) (Supplemental Table 1). Hence, variants in more than 200 genes may affect the AMG pathway. Since our findings indicated that seizures can be caused by 2 heterozygous LoF variants in different genes affecting the AMG pathway, it is possible that digenic pathogenic variants that affect actin polymerization, mitochondrial ROS, and/or glutamatergic transmission cause a subset of seizures. We therefore gathered an undiagnosed cohort of 433 individuals with seizures and 2,167 individuals without seizures from Baylor Genetics and analyzed their sequencing data by ranking the top risk variants using AI-MARRVEL, a comprehensive tool to prioritize possible disease-causing variants (77) (Figure 8A). Among the top-10 variants of each individual, the percentage of individuals with 2 or more LoF variants from AMG genes was significantly higher in the seizure cohort (5.5%) when compared with the nonseizure cohort (1.2%) (Figure 8B). We also downloaded and analyzed the sequencing data for 774 unaffected individuals as nonpatient controls from the 1000 Genomes Project dataset (78). The percentage of control individuals with 2 or more LoF variants in the AMG pathway genes was extremely low (<0.3%) (Supplemental Figure 14).

We selected 16 individuals with digenic LoF variants in genes known to regulate either actin polymerization or ROS (Supplemental Table 3). We evaluated the effects of the affected orthologous genes in flies for 7 of these individuals (Table 2 and Supplemental Figure 15) and observed that 5 of the 7 transheterozygous mutants had synergistic or additive effects with respect to heat sensitivity, suggesting that combining these alleles had a pathological consequence (Figure 8, C–G). In the remaining 2 digenic combinations, we did not observe obvious synergistic or additive effects (Supplemental Figure 15, A–D).

To assess whether digenic LoF variants cause spontaneous seizures, we recorded the behaviors of the flies for 4 days using a video-tracking system (79). As a negative control, we used *w^1118^* flies, as they do not exhibit seizures. As expected, we observed spontaneous seizures in *VGlut>sif* RNAi flies (Supplemental Figure 16, A and B). Next, we video-tracked 9 combinations of transheterozygous mutant flies including the 5 that modeled the individuals in Figure 8. As a result, we detected seizure-like events in 7 of the 10 genotypes at 25°C (Supplemental Figure 16A). We then recorded 4 of the genotypes at 29°C and observed spontaneous seizures (Supplemental Figure 16B and Supplemental Videos 4–7). These data show that digenic variants in genes that regulate the AMG pathway can promote seizure susceptibility.

## Discussion

Disruptions in the regulation of actin polymerization have been implicated in a variety of neurological diseases, including epilepsy (10, 11). We previously discovered that LoF variants in *TIAM1* are associated with neurodevelopmental phenotypes and seizures (16). Our in vitro and in vivo data reveal a critical role of *TIAM1* (fly *sif*) in mitochondrial distribution and function. The loss of *TIAM1/sif* led to a significant increase in Drp1 levels and activity (*P* = 0.02), which was associated with an increase in the number of mitochondria and a decrease in the size of mitochondria as well as elevated ROS levels. These features were associated with an increase in glutamatergic transmission and seizures. Moreover, we show that inhibition of mitochondrial fission using a Drp1 inhibitor, Mdivi-1, significantly (*P* = 0.003) suppressed seizure-like behaviors in *sif* mutants. Treating *sif* mutants with the BBB-penetrant antioxidant NACA also alleviated seizure-like behaviors and suppressed the increase in glutamatergic transmission observed in mutants. In addition, we show that double-heterozygous LoF alleles that affect the AMG pathway can additively or synergistically induce seizures, suggesting that in a subset of individuals with epilepsy, there is a digenic origin. In summary, we identified an AMG pathway that plays an important role in the pathogenesis of epilepsy.

The seizures in larvae originated in the CNS. Indeed, the amplitude and frequency of spontaneous EPSPs in muscles were not altered in larvae when the motor neurons were severed from the CNS (Supplemental Figure 4). In contrast, the EPSPs were increased in mutant muscles when the neurons were connected to the CNS (Figure 2). Hence, the NMJ hyperactivity was the result of the increased activity of neurons in the CNS that drove the activity of motor neurons, in agreement with our observation that the glutamatergic neuronal activity was increased in both the brain lobes and the VNC when *sif* RNAi was expressed (Supplemental Figure 2).

Mutations in actin regulatory genes are often associated with rare human neurodevelopmental diseases in which seizures are a prominent feature (10, 11). Bioinformatics queries revealed that approximately 50 epilepsy-associated genes affect actin polymerization, including *TIAM1* (16). These observations strongly suggest that actin dysregulation is associated with epilepsy, but the mechanisms have not been explored. We show that *TIAM1/sif* loss led to aberrant small actin clusters in the cell body of neurons that had previously only been observed in yeast (42). These small clusters are consistent with reduced actin polymerization levels. In addition, our genetic interaction studies show that heterozygous loss of epilepsy-associated actin regulatory genes interacted with *Profilin 1* (*PFN1/chic*) to increase bang sensitivity. These data indicate that reduced actin polymerization levels can cause seizures and that transheterozygous loss of some of these genes is associated with seizures.

Epilepsy commonly accompanies mitochondrial disorders (80), affecting 35%–60% of the patients, including those with Leigh syndrome (81), mitochondrial encephalopathies, myoclonic epilepsy with ragged-red fibers (MERRF), and others (82). How these alterations in mitochondrial function cause epilepsy is not well established. Our data indicate that, in a subset of individuals, these alterations may result from the dysregulation of actin dynamics, given that lack of *TIAM1/sif* caused elevated levels of Drp1 and mitochondrial fission. Short actin filaments are associated with mitochondria and recruit Drp1 (53, 55). Drp1 mediates mitochondrial fission (60, 83), and an increase in mitochondrial fission can increase ROS (66, 67). Interestingly, an increase in mitochondrial fission is necessary but is not sufficient to induce mitophagy (84). Moreover, recent studies have shown that Pink1/Parkin-mediated mitophagy requires actin polymerization surrounding the mitochondria (85). Given that loss of *DNM1L* (which encodes DRP1) also causes an increase in ROS (86, 87), and that suppression of ROS with NACA is beneficial (Figure 6), ROS is likely to be an important player in the pathogenesis of some forms of epilepsy. Moreover, the presented genetic interaction data provide further evidence that actin regulatory genes may cause seizures via ROS, indicating that this may not be an uncommon pathway. Importantly, the lack of additivity or synergy of the drugs used in this study indicates that mitochondrial fission and ROS affect the same pathway, and that both drugs may ameliorate these forms of epilepsy.

An increase in mitochondrial ROS in neurons has previously been shown to alter the trafficking of glutamate receptor subunits. Elevated ROS increases the surface expression of AMPA and NMDA receptors, leading to an increase in glutamatergic transmission in frontotemporal dementia (88). In *Drosophila*, an increase in glutamatergic transmission has also been shown to further increase ROS, leading to a feed-forward loop and seizures (23). Increased ROS has also been observed in *Sod2^+/–^* mice, and these animals have an increased susceptibility to seizures (89). Similarly, we found that *sod1* heterozygous flies (*sod1^T2A-GAL4^/+*) had mild bang sensitivity. These data are consistent with our observation that ROS drove an increase in glutamatergic transmission and that inhibiting ROS suppressed seizure frequency by reducing hyperexcitability in glutamatergic neurons. In addition, it has been reported that Mdivi-1 can act independently of Drp1 activity as a reversible inhibitor of mitochondrial respiration and can protect cells by reducing ROS in *Drp1*-KO cells (90, 91). Hence, Mdivi-1 may have a better rescue ability than *Drp1* RNAi. However, given that neither Mdivi-1 nor NACA fully rescued the seizure-like phenotypes in *sif* mutants, there are probably other contributors besides ROS to epileptogenesis. The incomplete rescues may be due to defects in cholinergic neurons, given that *sif* RNAi in cholinergic neurons led to severe climbing defects as well as seizure-like phenotypes (Figure 1, B–D), as loss of *sif* was recently shown to affect the function of cholinergic synapses (92). In addition, the activation of glial IIR was recently identified as another contributor to epilepsy (70). Our observations also show that glial IIR was activated in *sif* mutants. Hence, the therapeutic effects of inhibiting both mitochondrial fission/ROS and glial IIR should be explored.

Given that numerous genes have been implicated in epilepsy, affected individuals are often sequenced to explore a possible genetic diagnosis, yet more than 50% of these individuals do not receive a genetic diagnosis (5, 6). Monogenic models alone may therefore not explain numerous undiagnosed epilepsies, suggesting that combinations of variants may play an important role. Here, we found that individuals with undiagnosed epilepsy had an increased burden of LoF variants in the AMG pathway genes when compared with controls. Importantly, we identified individuals with seizures who carry 2 heterozygous LoF variants in genes affecting the AMG pathway based on sequencing, artificial intelligence–powered (AI-powered) prioritization and fly modeling. For 5 of 7 cases, heterozygous combinations of these variants had synergistic or additive effects resulting in seizures in flies. There are obviously many more individuals with variants in 2 genes that affect the AMG pathway, as we only modeled LoF variants. Other variant combinations will be the subject of future studies, as this approach may provide proof of concept for the diagnosis of individuals with digenic etiologies underlying epilepsy.

## Methods

### Sex as a biological variable.

Our study examined male and female animals, and similar findings are reported for both sexes. However, it is worth noting that male flies are generally more sensitive to heat than female flies.

Additional details on methods are provide in the Supplemental Methods.

### Drosophila stocks and maintenance.

All fly strains used in this study were obtained from the Bloomington Drosophila Stock Center (BDSC), received as gifts, or generated in the Bellen laboratory, and their genotypes are listed in Supplemental Table 4. The fly lines used for genetic interactions are listed in the genotypes section of Supplemental Table 2. Flies were raised and maintained at 25°C, unless otherwise specified in the text.

### Human neuronal cells.

Daoy cells (American Type Culture Collection [ATCC], HTB-186) were maintained in DMEM (Gibco, Thermo Fisher Scientific, 10-569-044) supplemented with 10% FBS and 1× penicillin and streptomycin. The *TIAM1* siRNA sequence was used previously (75), and the control siRNA was designed by VectorBuilder (Supplemental Table 5). Lipofectamine RNAiMAX (Invitrogen, Thermo Fisher Scientific, 13778075) was used to transfect the cells with siRNA at a final concentration of 50 nM. The cells were collected 72 hours after transfection for immunostaining or protein extraction.

### Drosophila behavioral assays.

The behavioral assays were carried out as described previously (16). Flies were allowed to climb for 20 seconds unless otherwise mentioned in the figure legend, and the climbing distance was measured (15 cm as the maximum). For the bang sensitivity assay, flies were vortexed at maximum speed for 10 seconds, and the time to recover to the freely moving status was measured. For the heat sensitivity assay, a group of 5 or more flies were submerged in a 42°C water bath for 30 seconds unless noted otherwise. The percentage of flies unable to keep an upright position was measured after heating. Recovery was assessed by measuring the time taken for the flies to recover to a coordinated movement (30 seconds as the maximum). The survival rate was measured using the expected Mendelian ratio by counting flies that eclosed and were viable after 24 hours.

### Electrophysiology and analysis.

Electrophysiology was performed as previously described (93). Third instar larvae raised at 25°C were dissected in the hemolymph-like solution 3 (HL3) solution without CaCl_2_. During recordings, the solution was changed to HL3 plus CaCl_2_ (1 mM), and EPSPs were recorded from muscle 7 of abdominal segments 3 and 4. Recordings from muscles with a resting membrane potential below –55 mV and an input resistance of greater than 4 mΩ were selected for analysis.

### Immunostaining.

Immunostaining of fly larval and adult brains was performed as described previously (94). Information on the antibodies used is provided in Supplemental Table 6. Images taken with a confocal microscope were analyzed using Fiji-ImageJ (NIH) and Imaris.

### Larval crawling assay.

Third instar larvae raised at 25°C were used to assess their crawling ability. Larvae were collected and transferred to a Petri dish with agar, and the crawling distance was measured for a 1-minute period.

### Drug treatment in flies.

Drug treatment was performed as previously described (95). For survival and behavioral phenotypes, flies were maintained on food containing NACA (MilliporeSigma, A0737) or Mdivi-1 (Tocris, 3982-50) or were given control food. Information on the chemicals used in this study is provided in Supplemental Table 6.

### TEM.

TEM was performed as previously described (96). The VNC of third instar larvae were prepared for TEM imaging. Data were analyzed using Fiji-ImageJ (NIH).

### Western immunoblotting.

Western immunoblotting of fly tissues (97) and mammalian cell samples (98) was performed to determine protein levels. Protein lysates from fly tissues or mammalian cells were denatured and loaded onto SDS-PAGE (Bio-Rad). All antibodies used are documented in Supplemental Table 6.

### Aconitase assay.

The aconitase assay was performed using an Aconitase Activity Assay kit (Abcam, ab83459) as previously described (96). Third instar larvae raised at 25°C were used for the assay. The absorbance was measured using a FLUOstar OPTIMA microplate reader (BMG Labtech).

### AI-MARRVEL analysis.

Baylor Genetics data were gathered from patients undergoing clinical exome or genome sequencing. Variants in the VCF format along with the Human Phenotype Ontology (HPO) terms for each individual were used as inputs for AI-MARRVEL (77) to prioritize genetic variants that were potentially associated with the individual’s disease phenotype. The prediction scores were used for downstream analysis. Seizure-related HPO terms were collected from the HPO database (99) through manual search and validation (Supplemental Table 7). For each individual, the top 10 most likely pathogenic variants were extracted according to AI-MARRVEL prediction scores across all variants identified in the individual. Subsequently, a curated actin-mitochondria-glutamate gene set (Supplemental Table 8) was used to identify patients with digenic variants in this gene set. The control cohort of 774 individuals is a random subset from the 1000 Genomes Project dataset (78). The sequencing data were analyzed using the same epilepsy-related HPO terms as the Baylor Genetics epilepsy cohort so that the prioritized genes reflect genomic signal rather than differences in phenotype input.

### Statistics.

Statistical analysis was performed using GraphPad 9.0 (GraphPad Software). Pairwise group comparisons were conducted using unpaired 2 tailed *t* tests (with Welch’s correction applied when variances were significantly different), unless otherwise specified in the figure legends. Data are presented as the mean ± SEM, and NS indicates *P* > 0.05.

### Study approval.

Clinical data for individuals were from Baylor Genetics. Minimum clinical information of the deidentified individuals and corresponding sequencing data were analyzed for the purpose of improving the diagnostics. This work was approved by the IRB at Baylor College of Medicine (IRB protocol H-41191, H-56038).

### Data availability.

All data associated with this study are present in this article or the supplemental materials. All data values in this work can be found in the Supporting Data Values file. Information on the software and algorithms used in this study can be found in Supplemental Table 9. The data and additional details on protocols used in this study are available from the corresponding author upon reasonable request.

## Author contributions

SL and HJB designed most experiments and wrote the manuscript. SL generated most of the data for this project. MM conducted many experiments and helped with analyses of the data. SBH and SL performed electrophysiological recordings, the live imaging, and data analyses. LDG performed aconitase assays, edited the manuscript and provided feedback. MD performed some *Drosophila* behavioral experiments, genetic studies and data analyses. HC, MD, ZY and SL performed genetic analyses of the undiagnosed epilepsy cohort under the supervision of ZL and HJB. HK performed video-tracking and relevant data analysis with SL under the supervision of VAC and HJB. YZ performed *Drosophila* behavioral experiments and data analyses of mammalian cells with SL. JAR and XL provided clinical information. WWL performed TEM imaging. SKD, XP and DD conducted some experiments, edited the manuscript, and provided feedback. JMS edited the manuscript and provided feedback.

## Conflict of interest

The Department of Molecular and Human Genetics at Baylor College of Medicine receives revenue from clinical genetic testing completed at Baylor Genetics Laboratories.

## Funding support

This work is the result of funding in part by the National Institutes of Health (NIH), and is subject to the NIH Public Access Policy. Through acceptance of this federal funding, the NIH has been given a right to make the work publicly available in PubMed Central.

- NIH grant U01 AG072439 (to HJB).
- Huffington Foundation grant (to HJB).
- Neurogenetics Chair of the Neurological Research Institute (NRI) (to HJB).
- NRI Zoghbi Scholar Award (to SL).
- NRI Zoghbi Scholar Award (to LDG).
- TNPO2 Foundation (to LDG).
- BrightFocus Foundation (to LDG).
- The Effie Marie Cain Chair in Alzheimer’s Disease Research at Baylor College of Medicine (to JMS).
- The Huffington Foundation Chair in Parkinson’s Disease Research at Texas Children’s Hospital (to JMS).
- German Research Foundation (to SBH).
- Walter Benjamin Fellowship (to SBH).
- Cancer Prevention and Research Institute of Texas award (CPRIT RP240131, to ZL and HC).
- This work was supported by infrastructure made available by the Texas Children’s NRI (NICHD P50-HD103555) and the Intellectual and Developmental Disabilities Research Center at Baylor College of Medicine (BCM) (U54HD083092 from the Eunice Kennedy Shriver NICHD) for Microscopy Core facilities.

## Supplementary Material

Supplemental data

Unedited blot and gel images

Supplemental tables 1-9

Supplemental video 1

Supplemental video 2

Supplemental video 3

Supplemental video 4

Supplemental video 5

Supplemental video 6

Supplemental video 7

Supporting data values

## Figures and Tables

**Table 1:**
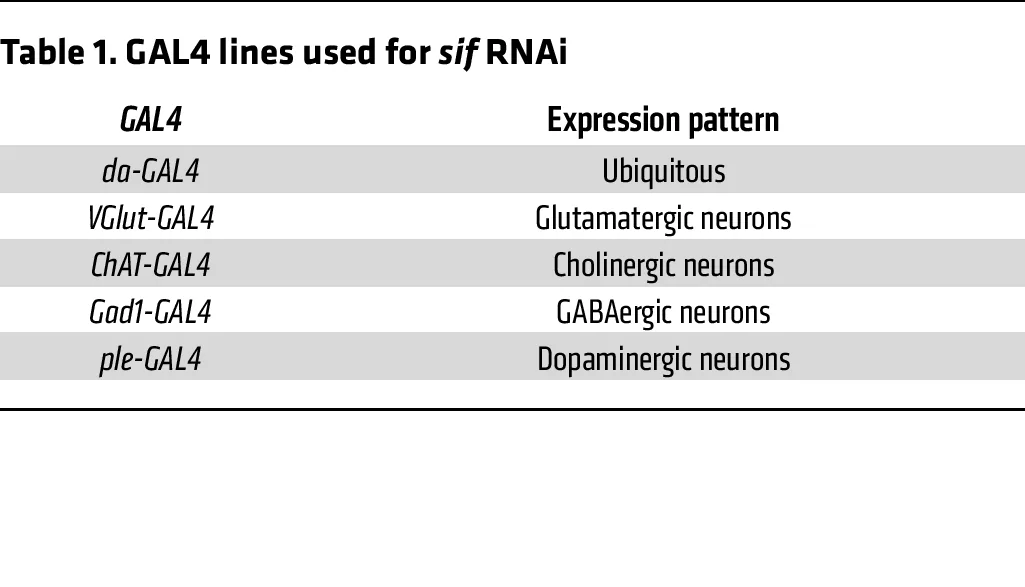
GAL4 lines used for *sif* RNAi

**Table 2:**
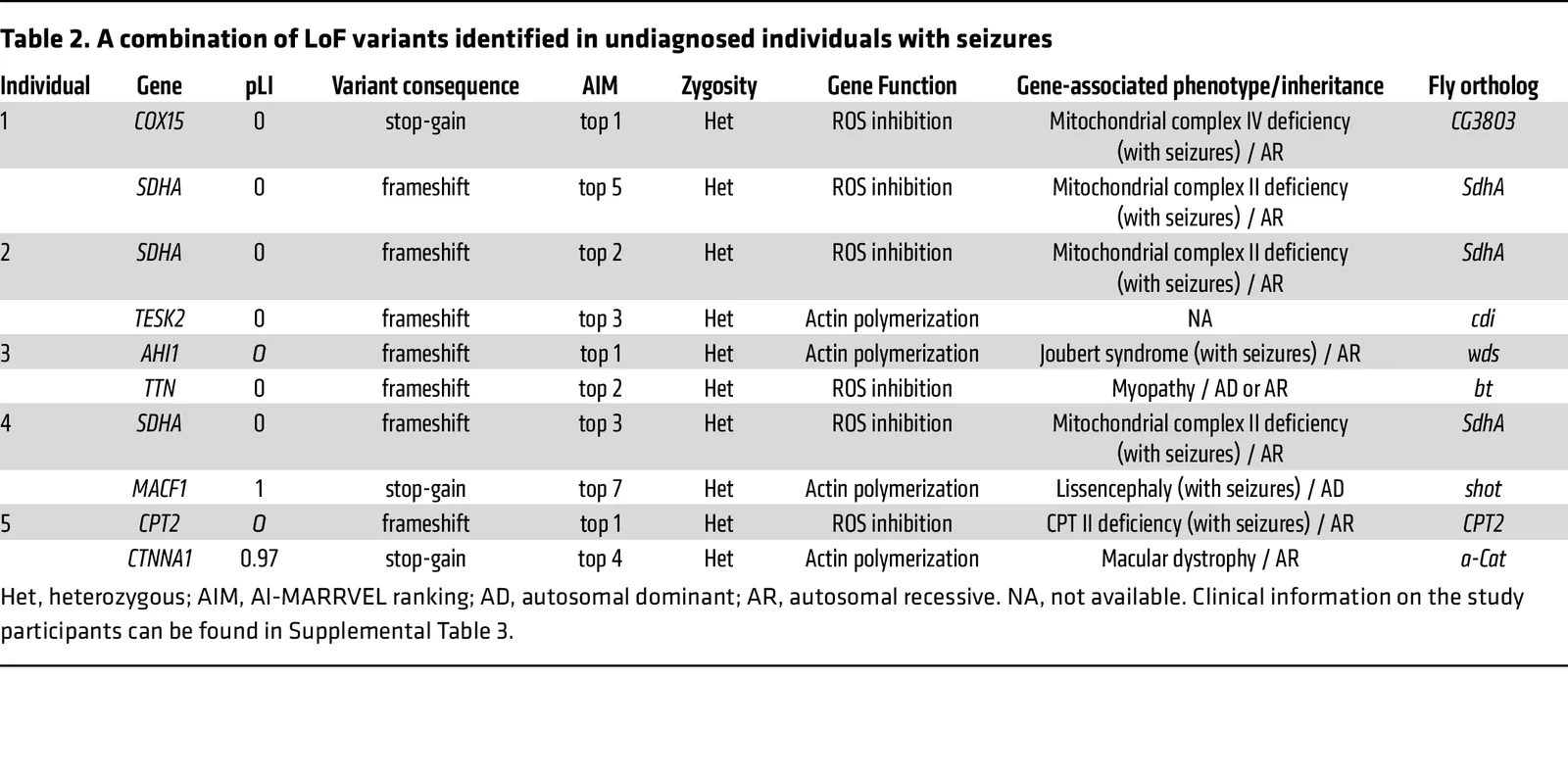
A combination of LoF variants identified in undiagnosed individuals with seizures

## Version 1 07/16/2026

In-Press Preview

## Version 2 09/01/2026

Electronic publication
