# Supplementary material for: Epilepsy-associated digenic variants affecting an actin/mitochondria/glutamate pathway promote seizure susceptibility
Source: J Clin Invest. 2026 Jul 16;136(17):e198696. doi: 10.1172/JCI198696 (PMC13528942; doi:10.1172/JCI198696)
Supplement: Supplementary file 1 [file jci-136-198696-s044.pdf]

Supplemental Methods

ATP determination. The ATP determination kit from Thermo Fisher Scientific (A22066) was used to measure ATP levels. Five larvae were homogenized in 50 µL of homogenization buffer, followed by boiling for 5 min and centrifugation at ~20,000g for 3-5 min at 4 °C. 10 µL of supernatant was collected for the measurement of ATP levels in the luminometer by following the protocol. Total protein levels were determined to normalize the data.

Real-time quantitative PCR (qPCR). Real-time qPCR was performed as previously described (1). All-In-One 5X RT MasterMix (abm, #G592), iTaq Universal SYBR Green Master Mix (BioRad, #1725120) and a BioRad C1000 Touch Cycler were used. Primer sequences (2) are listed in Supplemental Table 5.

Mitochondrial ROS measurement using the UAS-mito-roGFP2-Orp1 reporter. Fly brains of each genotype were dissected at 3-5 days in Artificial Hemolymph (AHL) solution with 20 mM N-ethyl maleimide (NEM, Sigma, E3876), a fast-acting roGFP2-thiol-blocking agent that prevents further probe oxidation or reduction (3, 4). Then the fly brains were fixed in 4% PFA, followed by confocal microscopy, with excitation sequentially at 405 or 488 nm and the emission range was held constant at 500-530 nm. The 405 and 488 ratio was measured and quantified.

Live imaging of fly brains. Fly brains were dissected at 3~5 days in cold Schneider's Medium (S2). To assess the mitochondrial membrane potential, the brains were incubated in 200 nM TMRM (Invitrogen, T668) in S2 medium at room temperature for 30 minutes and brains were mounted in 50 nM TMRM in PBS. Confocal images were taken immediately (Excitation/Emission: 548/574 nm). To assess mitochondrial ROS levels, fly brains were incubated in 5 µM MitoSOX (ThermoFisher, M36007) in S2 medium at room temperature for 30 minutes. Then the fly brains were washed in PBS for 5 minutes and mounted in PBS. Confocal images were taken immediately (Excitation/Emission: 396/610 nm).

Video-tracking of flies. Spontaneous seizures were detected as previously described (5). Flies were placed into individual wells of a 48-well plate containing 1% agarose and 5%

sucrose. Flies were video-recorded using an infrared camera at 25 frames per second at 25 °C or 29 °C for 4 days in a 12 hour light:12 hour dark cycle with an IR backlight. Noldus EthoVision XT was used to extrapolate XY coordinates over time from videos. Custom MATLAB scripts (CynthiSeize v1.3) were used to identify seizures; all scripts, instructions, and a sample dataset with outputs are available at https://zenodo.org/records/15619740. A threshold of 10 hyperkinetic events within 50 seconds was used to define seizures, with a minimum inter-seizure interval of 15 minutes. All seizures detected are reported without manual scoring or screening. The hyperkinetic seizures are indicated by red arrows within the videos.

Videos

Video 1. Bang sensitivity assay of trans-heterozygous flies. Left vial: *Rac1 chic* trans-heterozygous flies; Right vial: *Rac2 chic* trans-heterozygous flies.

Video 2. Bang sensitivity assay of *SWIP chic* trans-heterozygous flies.

Video 3. Bang sensitivity assay of *cher chic* trans-heterozygous flies.

Video 4. Spontaneous seizure activity in *VGlut>sif RNAi* flies. The *VGlut>sif RNAi* fly in Well #16 (white arrow) shows a spontaneous seizure event. Hyperkinetic movements characteristic of seizures are highlighted with red arrows. Control (*w1118*) flies were also labeled (Wells #17-#48) in the video. Note that the flies that do not move are typically sleeping.

Video 5. Spontaneous seizure activity in *cher* and *Eaat2* trans-heterozygous flies. The *cher*^{MiMIC}/+; *Eaat2*$^{T2A\text{-}GAL4}$ trans-heterozygous fly in Well #33 (white arrow) exhibits a seizure. Hyperkinetic movements characteristic of seizures are highlighted with red arrows.

Video 6. Spontaneous seizure activity in *Rac2* and *chic* trans-heterozygous flies. The *Rac2*^{KG05681} /+; *chic*^{01320}/+ trans-heterozygous fly in Well #3 (white arrow) exhibits a spontaneous seizure. Hyperkinetic events characteristic of seizures are highlighted with red arrows.

Video 7. Spontaneous seizure activity in *CPT2* and *α-Cat* trans-heterozygous flies. The *CPT2*^{KG05681}/+; *a-Cat*^{MiMIC}/+ trans-heterozygous fly in Well #2 (white arrow) exhibits a seizure. Hyperkinetic events characteristic of seizures are highlighted with red arrows.

References

1. Lu S, Ma M, Mao X, Bacino CA, Jankovic J, Sutton VR, et al. De novo variants in FRMD5 are associated with developmental delay, intellectual disability, ataxia, and abnormalities of eye movement. *Am J Hum Genet.* 2022;109(10):1932-43.
2. Nukala KM, Lilienthal AJ, Lye SH, Bassuk AG, Chtarbanova S, and Manak JR. Downregulation of oxidative stress-mediated glial innate immune response suppresses seizures in a fly epilepsy model. *Cell Rep.* 2023;42(1):112004.
3. Albrecht SC, Barata AG, Grosshans J, Teleman AA, and Dick TP. In vivo mapping of hydrogen peroxide and oxidized glutathione reveals chemical and regional specificity of redox homeostasis. *Cell Metab.* 2011;14(6):819-29.
4. Haynes PR, Pyfrom ES, Li Y, Stein C, Cuddapah VA, Jacobs JA, et al. A neuron-glia lipid metabolic cycle couples daily sleep to mitochondrial homeostasis. *Nat Neurosci.* 2024;27(4):666-78.
5. Cuddapah VA, Hsu CT, Valle Sirias F, Li Y, Shah HM, Saul C, et al. Sleep drive, not total sleep amount, increases seizure risk. *Nat Commun.* 2025;16(1):6967.

A

Actin regulatory genes known to be associated with epilepsy

Gene	Protein Function	Phenotype(s)
ACTB	Beta-actin	MCD
ACTG1	Gamma-actin	MCD
APC2	Actin organization	DEE, MCD
CAPZA2	F-actin-capping	DEE
CCDC88A	Actin-binding	DEE
CCT3	Actin-folding	DEE, MCD
CCT6A	Actin-folding	DEE, MCD
CTNNA2	Actin-binding	DEE, MCD
CYFIP2	Actin regulation	DEE
DAG1	Actin regulation	MCD
DIAPH1	Actin organization	DEE
FLNA	Actin crosslinking	MCD
FRMD5	Actin organization	DEE
HAX1	Actin reorganization	DEE, MCD
KPTN	Actin-binding	DEE
MACF1	Actin crosslinking	DEE, MCD
MTOR	Actin rearrangement	DEE, MCD
NCKAP1	Actin organization	DEE
OCRL	Actin polymerization	DEE
PAK1	Actin organization	DEE
PAK2	Actin organization	MCD
PAK3	Actin organization	DEE
PHACTR1	Actin interaction	DEE
PI4K2A	Actin organization	DEE
RAC1	Actin polymerization	DEE
RAC3	Actin polymerization	DEE
SPTAN1	Actin crosslinking	DEE
SPTBN1	Actin binding	DEE
SPTBN4	Actin interaction	DEE
SYNE1	Actin crosslinking	PME
TIAM1	Actin polymerization	**DEE**
WASF1	Actin organization	DEE
WASHC4	Actin polymerization	DEE
… …	… …	… …

B

Actin regulatory pathways in neurons

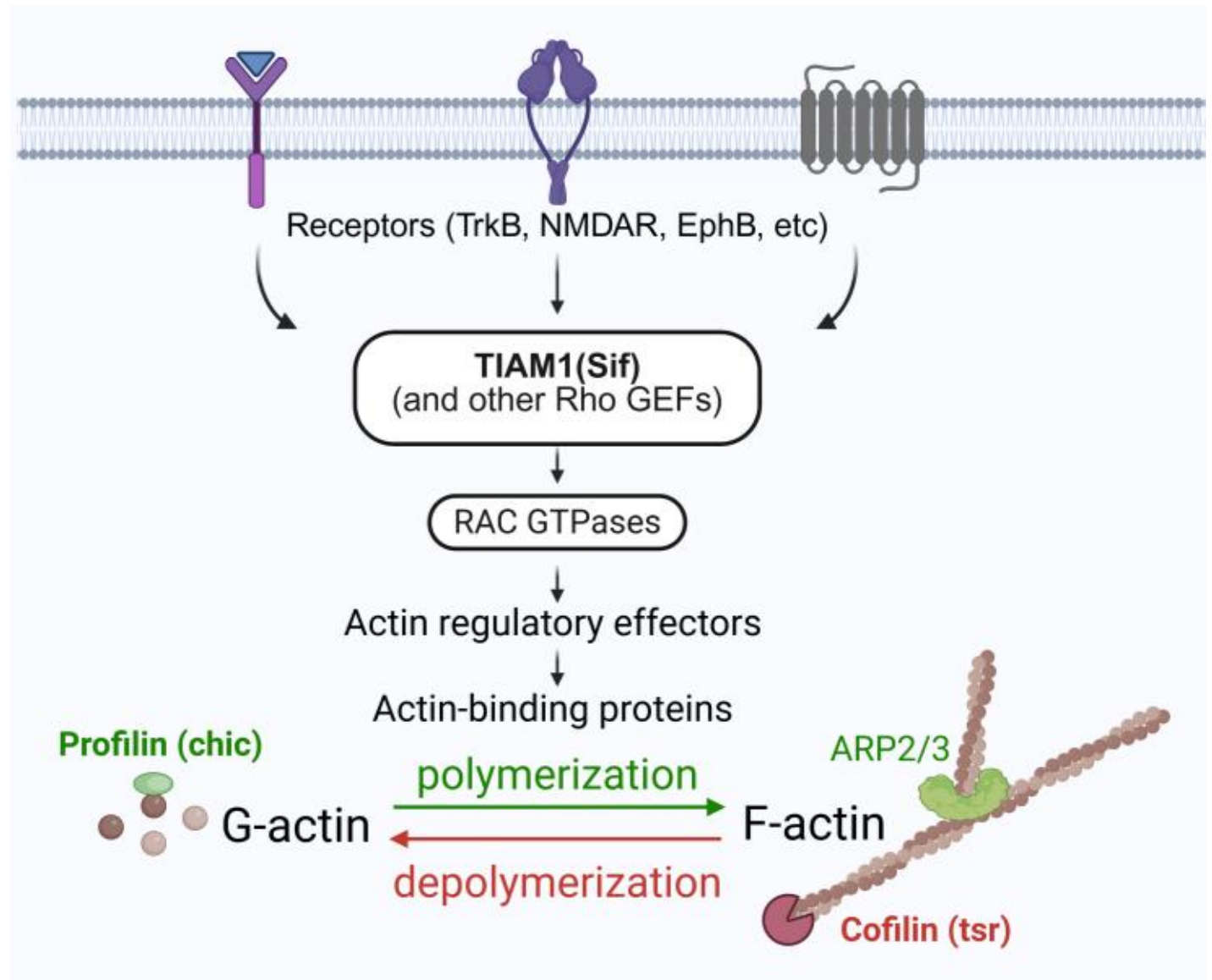

Supplemental Figure 1. Actin regulatory genes are associated with epilepsy. (**A**) List of actin regulatory genes known to be associated with epilepsy. Genes were identified by querying OMIM and reviewing the literature. DEE, developmental and epileptic encephalopathy. MCD, malformations of cortical development. PME, progressive myoclonus epilepsy. A comprehensive gene list is provided in Supplemental Table 1. (**B**) TIAM1 is a guanine nucleotide exchange factor (GEF) that regulates actin polymerization by activating RAC GTPase activity. The downstream effectors of RAC GTPases regulate actin dynamics together with actin-binding proteins. Panel B was created with BioRender.

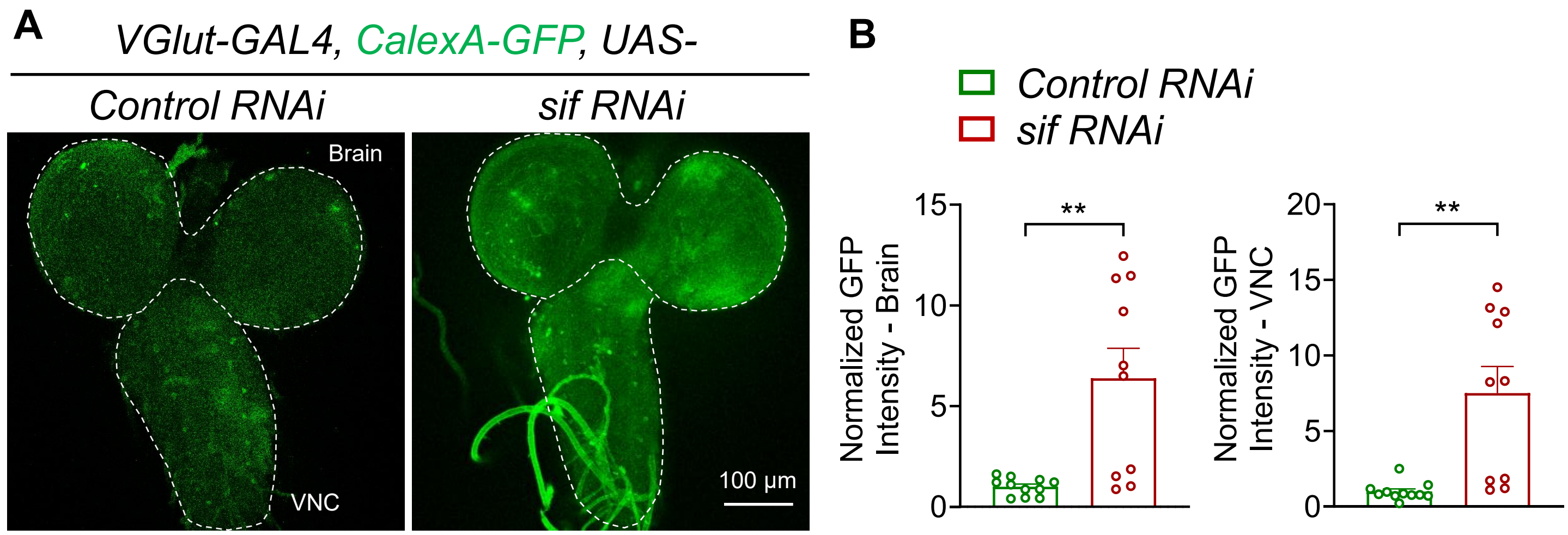

Supplemental Figure 2. Increased activity of glutamatergic neurons in both brain lobes and VNC after *sif* RNAi. The CaLexA-GFP system was used to label active neurons in specific neuronal populations. (**A**) The central nervous system (CNS) of the third instar larvae from *VGlut-GAL4 > luciferase RNAi* (control RNAi) and *VGlut-GAL4 > sif RNAi* animals was stained with an anti-GFP antibody. (**B**) The GFP signals were measured and quantified (n = 10-11). Data are presented as mean + SEM.

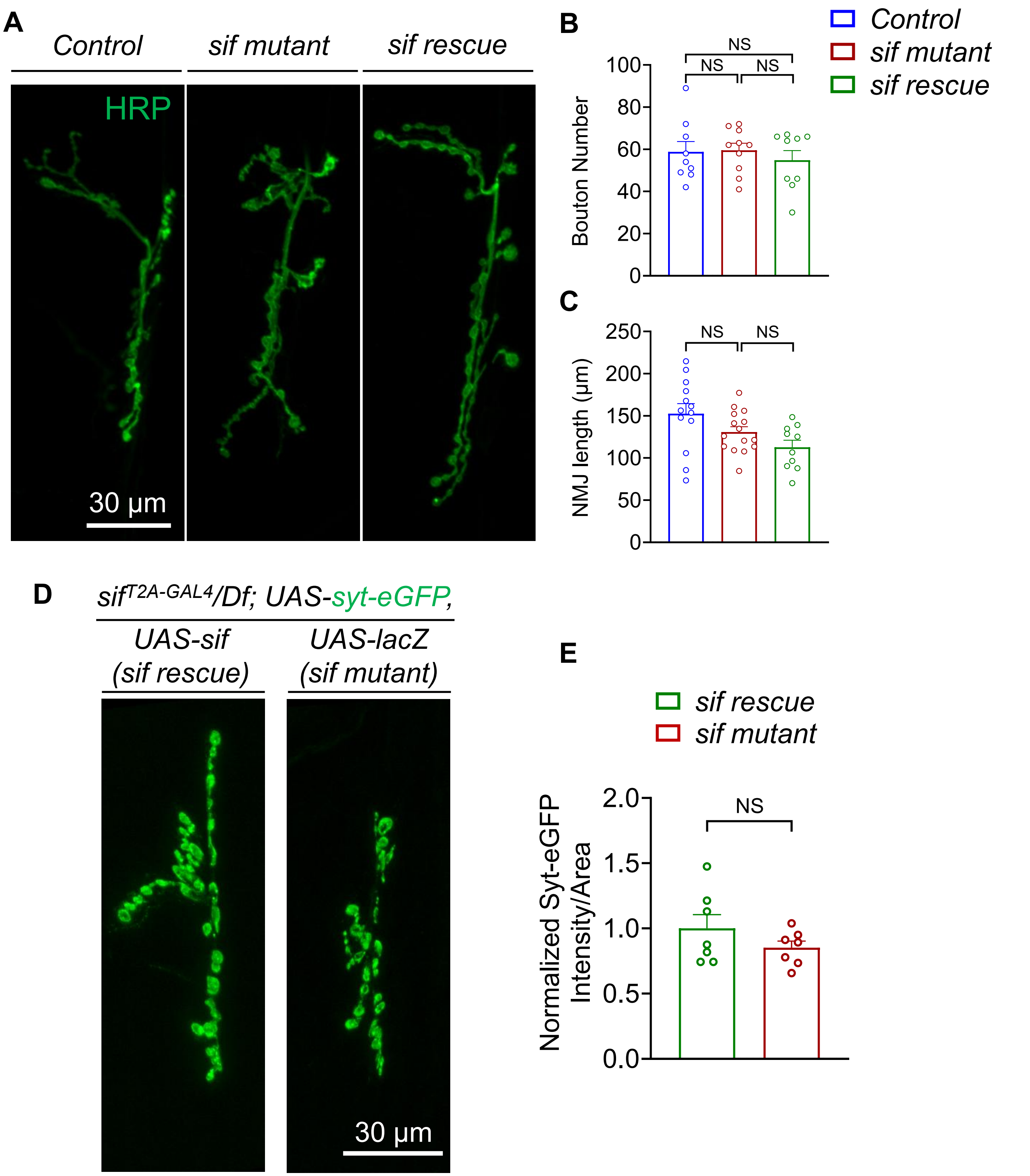

Supplemental Figure 3. *sif* loss does not obviously affect the NMJ morphology. (**A-C**) HRP staining of motoneurons at neuromuscular junctions (NMJs) does not reveal obvious differences in bouton number (B) or NMJ length (C) (n = 9-15). (**D-E**) Synaptic vesicles visualized with Syt-eGFP (D). The Syt-eGFP signal is not significantly altered in *sif* mutant larvae (E) (n = 7). Genotypes: *Control*: w^{1118}, *sif mutant*: *w; UAS-lacZ/+; sif*$^{T2A\text{-}GAL4}$*/Df*, *sif rescue*: *w; UAS-sif/+; sif*$^{T2A\text{-}GAL4}$*/Df.* Third instar larvae were used for the assays. Data are presented as mean + SEM.

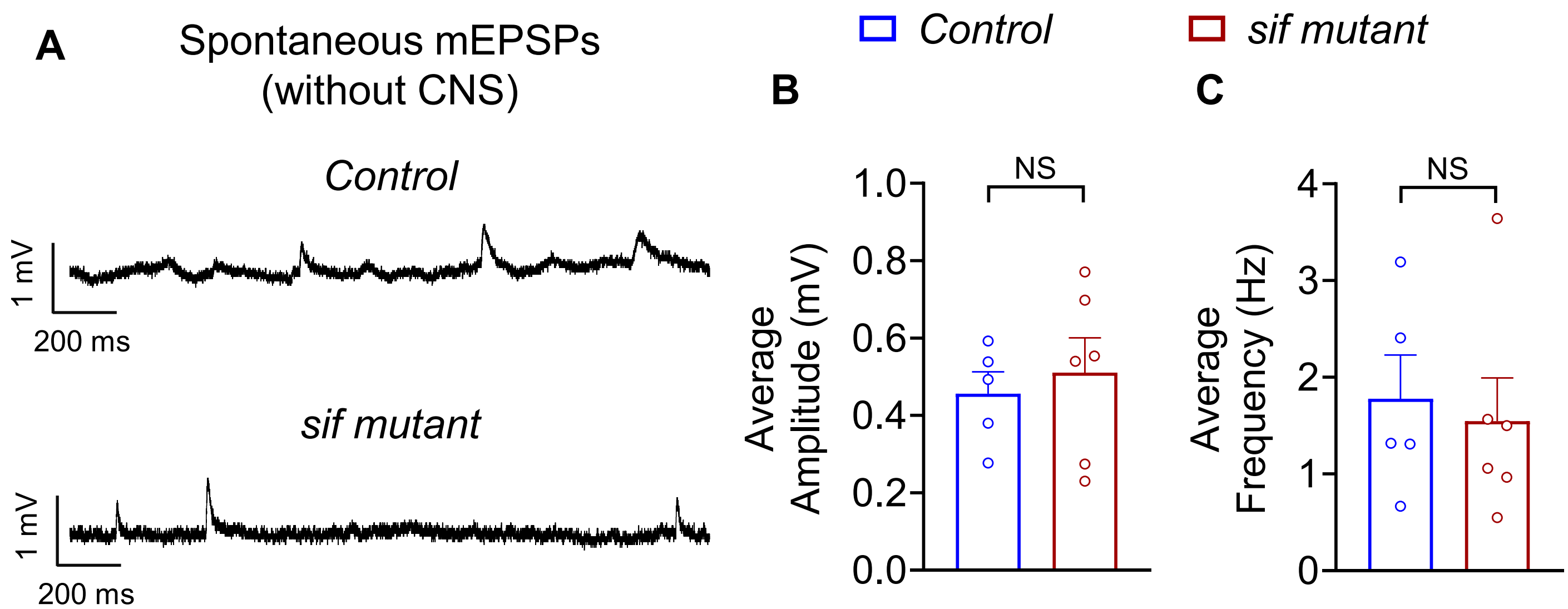

Supplemental Figure 4. No obvious changes in spontaneous mEPSP properties of NMJs without CNS upon *sif* loss. (**A-C**) Representative electrophysiological traces of spontaneous activity of control and *sif* mutant larval NMJs after severing the nerves from the CNS (A). Average spontaneous mEPSP amplitude (B) and frequency (C) are quantified (n = 5-6). Genotypes: *control* (*w^{1118}*), *sif mutant* (*w; UAS-lacZ/+; $sif^{T2A\text{-}GAL4}$/Df*). Third instar larvae were used for the assay. Data are presented as mean + SEM.

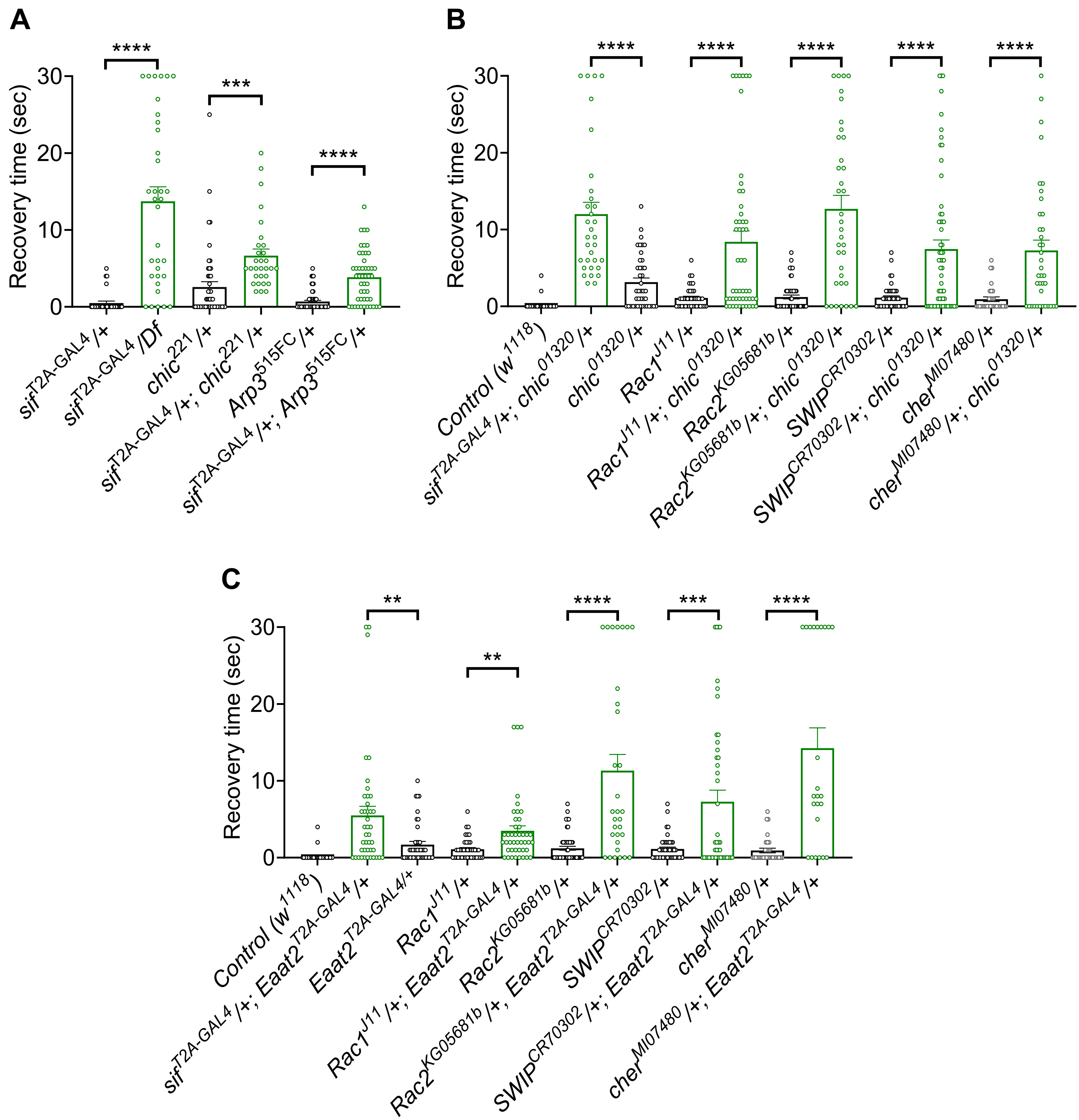

Supplemental Figure 5. Bang sensitivity of mutants in genes associated with actin regulation and glutamatergic transmission. (**A**) *sif* LoF allele synergizes with *PFN1* (*chic*) and *ARP3* (*Arp3*) alleles to cause bang sensitivity (n = 30-58). (**B**) Genetic interaction assays of *chic* and other actin regulatory genes (n = 30-60). (**C**) Genetic interaction assays of *EAAT2* (*Eaat2*) and other actin regulatory genes (n = 24-55). Flies were tested at 3-6 days post-eclosion (dpe). Data are presented as mean + SEM.

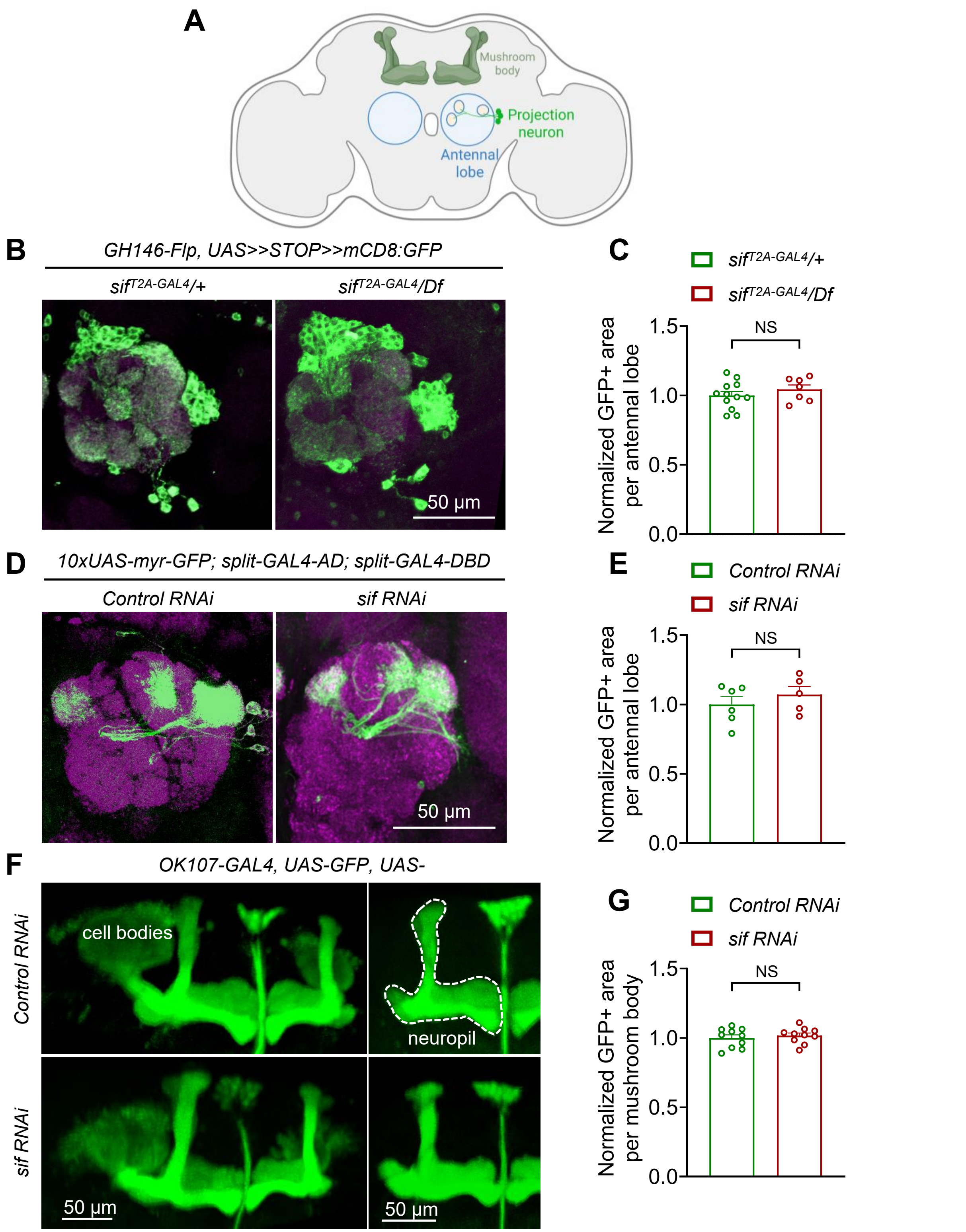

Supplemental Figure 6. No obvious neural wiring defects in the projection neurons and mushroom body with *sif* loss. (**A**) Schematic of the fly mushroom body and antennal lobe created with BioRender. Specific projection neurons (green) target dendrites to distinct glomeruli in the antennal lobe (blue). (**B-C**) *GH146*+ and *sif*+ projection neurons were genetically labeled with mCD8:GFP (B), and the GFP+ area per antennal lobe was quantified (C) (n = 7-12). (**D-E**) A *split-GAL4* driven *myr-GFP* was used to assess a subgroup of projection neurons with control RNAi or *sif* RNAi (D). The GFP+ area per antennal lobe was quantified (E) (n = 5-6). Magenta, N-cadherin. (**F-G**) The *OK107-GAL4* driven GFP was used to label the mushroom body neurons with control RNAi or *sif* RNAi. The GFP+ area per mushroom body neuropil was quantified (G) (n = 10). Fly brains were dissected at 3-5 dpe. Data are presented as mean + SEM.

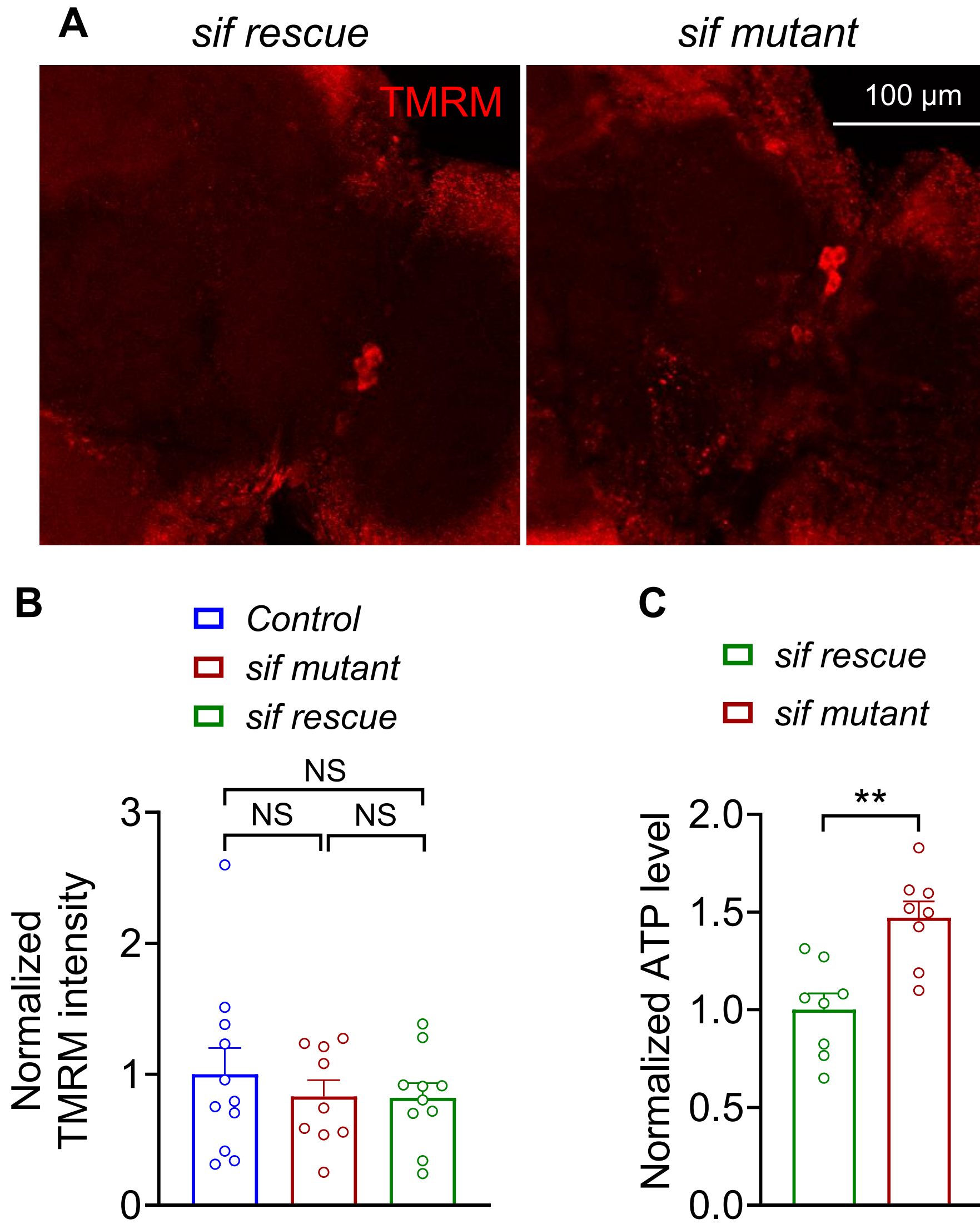

Supplemental Figure 7. Mitochondrial membrane potential is not obviously changed in the *sif* mutant brain. (**A**) The membrane potential was measured using TMRM (Tetramethylrhodamine, methyl ester) staining. Brains from *control* (w^{1118}), *sif mutant*, and *sif rescue* flies were dissected at 5 dpe, followed by TMRM staining and microscopy. (**B**) The TMRM intensity was measured and quantified (n = 9-11). (**C**) ATP levels of fly heads were assessed for *sif rescue* and *sif mutant* animals. Each datapoint represents the relative normalized ATP level per independent repeat (n = 8). Fly heads were collected at 3-5 dpe. Data are presented as mean + SEM.

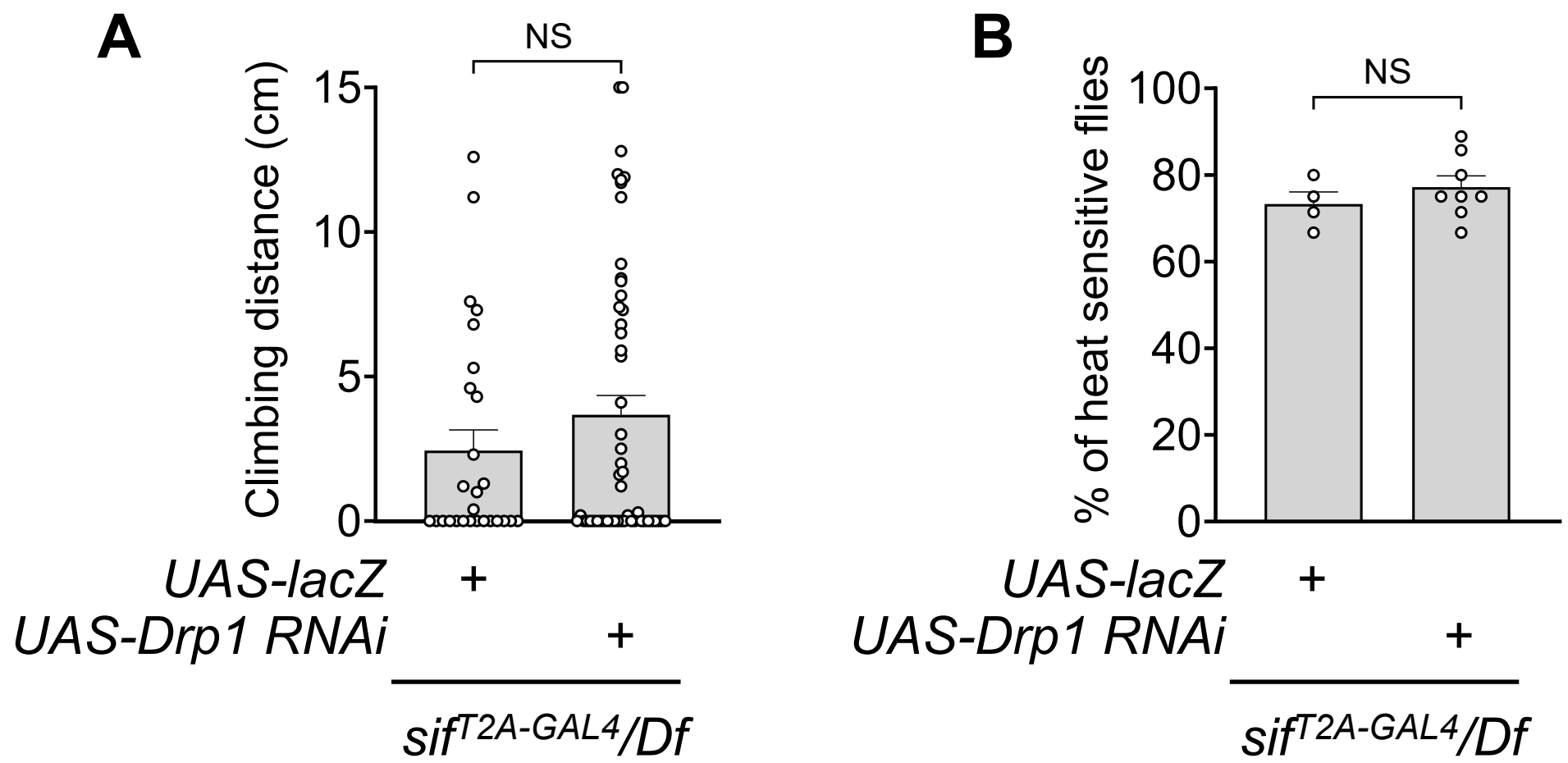

Supplemental Figure 8. *Drp1* RNAi does not rescue the climbing defects and bang sensitivity of *sif* mutant flies. (**A**) Flies were tapped to the bottom of the cylinder and allowed to climb for 1 minute, and the vertical distance of each fly was measured (maximum 15 cm) (n = 27-52). (**B**) Flies were exposed to 42 °C heat for 30 seconds and the heat-sensitive flies were counted (n = 4-8). Flies were tested at 2-4 dpe. Data are presented as mean + SEM.

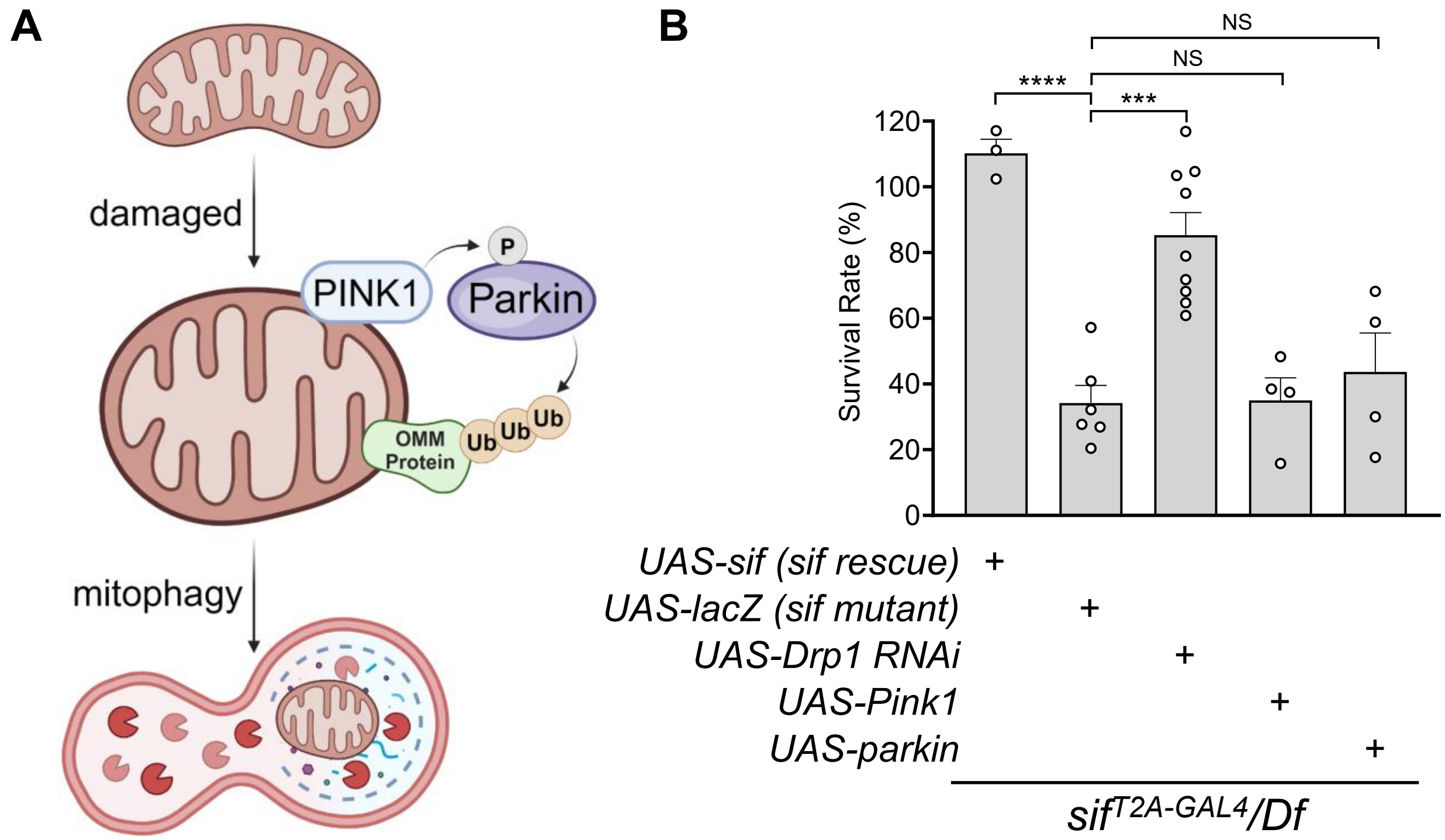

Supplemental Figure 9. Increasing expression of genes that induce mitophagy in *sif* mutants does not alter survival rate. (**A**) Model of PINK1-Parkin-induced mitophagy created with BioRender. PTEN-induced putative kinase protein 1 (PINK1) accumulates and recruits the E3 ubiquitin ligase Parkin to damaged mitochondria. Parkin ubiquitylates outer mitochondrial membrane (OMM) proteins to induce mitophagy. (**B**) Quantification of the survival rate of *sif* LoF mutants with expression of *UAS-Pink1* or *UAS-parkin.* The survival rate was measured using the expected Mendelian ratio by counting the survived flies that eclosed after 24 hours (n = 3-9). Data are presented as mean + SEM.

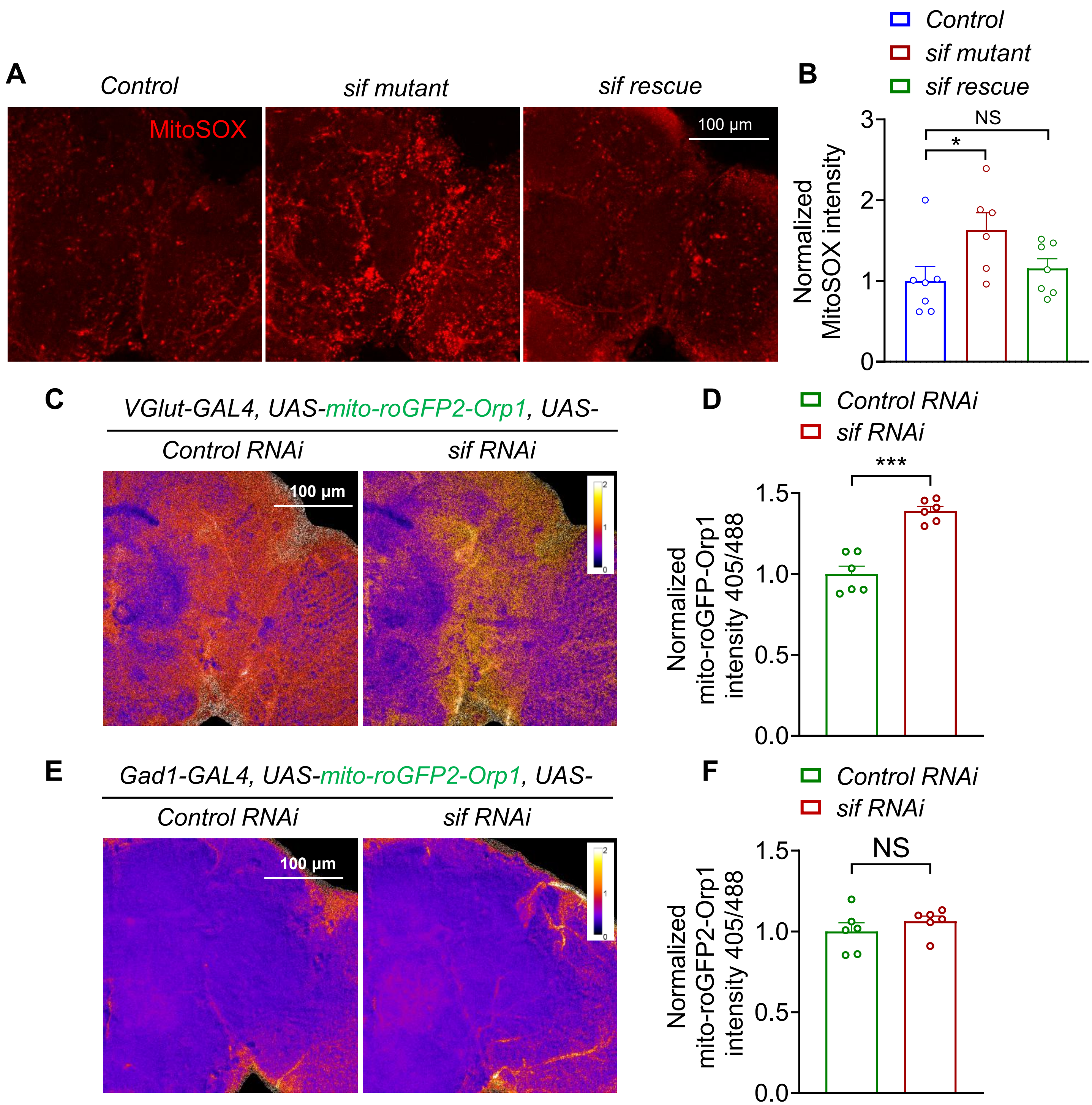

Supplemental Figure 10. Increased mitochondrial ROS levels in sif LoF fly brains, but not GABAergic *sif* RNAi fly brains. (A) The mitochondrial ROS levels were measured by MitoSOX staining. Brains from control (w^{1118}), *sif mutant*, and *sif rescue* flies were dissected at 5 dpe, followed by staining and live imaging. (**B**) The MitoSOX signals were measured and quantified (n = 6-7). (**C-F**) The mito-roGFP2-Orp1 is used to measure the H_2O_2 levels in the mitochondrial matrix. Brains from control RNAi and *sif* RNAi flies at 3-5 dpe were dissected and fixed, followed by confocal microscopy. The representative brains were false-colored with the fire LUC. The 405 and 488 ratio was measured and quantified (n = 6). Data are presented as mean + SEM.

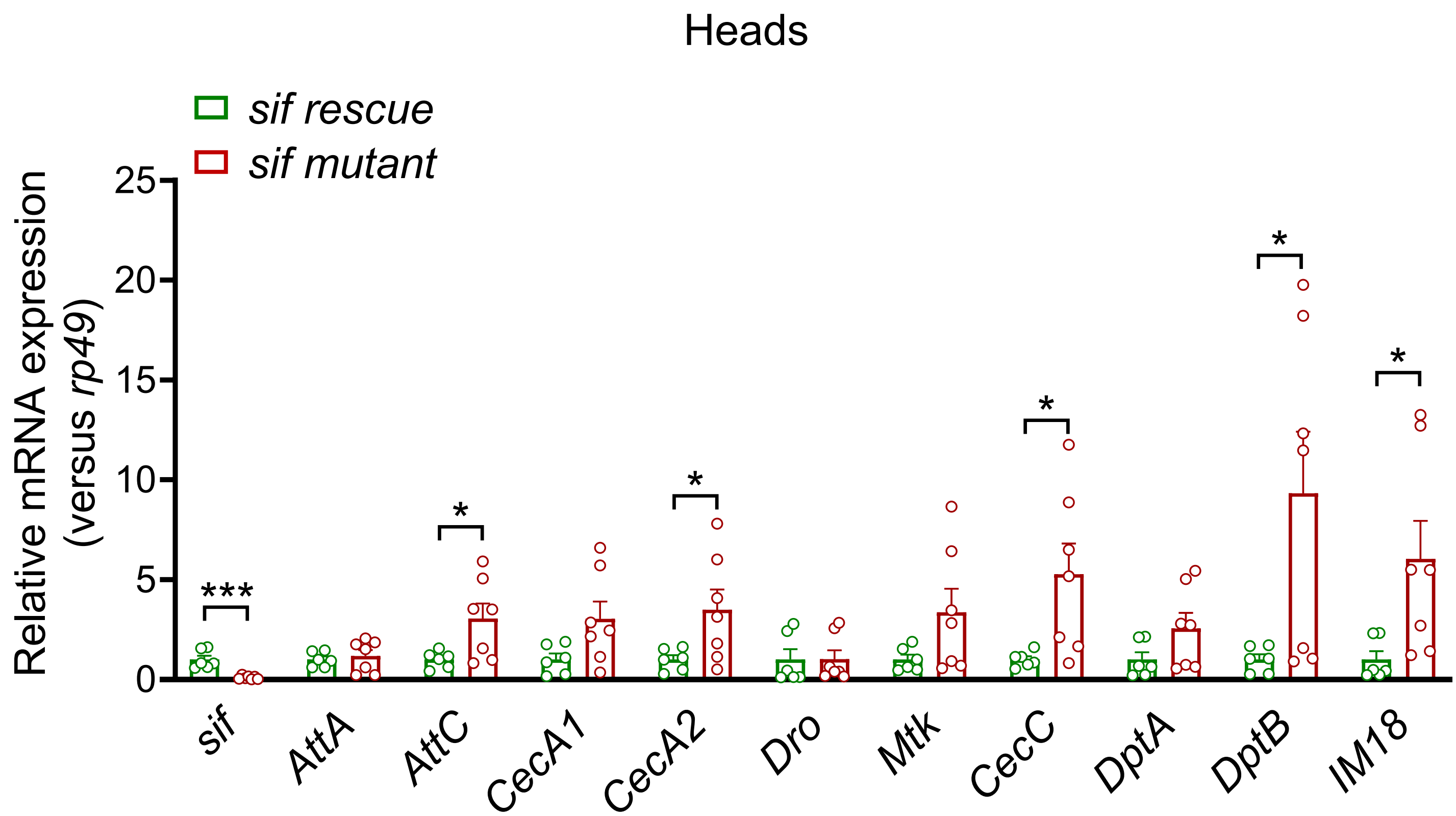

Supplemental Figure 11. Significant upregulation of innate immune response pathway genes in *sif* mutant heads. qRT-PCR analysis showing the relative mRNA expression levels of specific anti-microbial peptide (AMP) genes in *sif* mutant heads compared with *sif rescue* fly heads (n = 6-7). Fly heads were collected at 8-12 dpe for the analysis. Data are presented as mean + SEM.

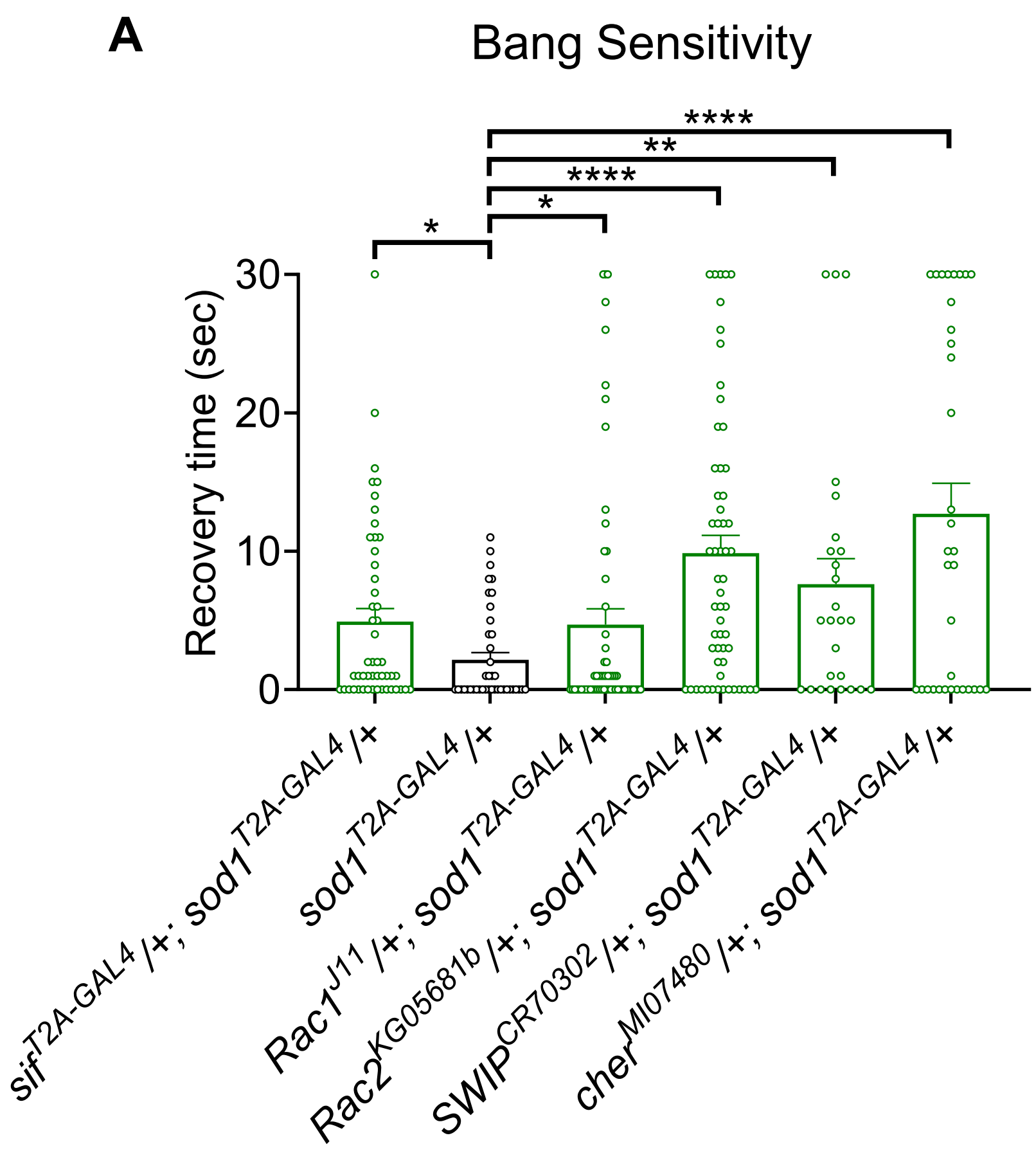

B Summary of genetic interactions (Trans-het: *GeneA*^{LoF}/+, *GeneB*^{LoF}/+)

Gene	*Profilin (chic)*	*EAAT2 (Eaat2)*	*SOD1 (sod1)*
TIAM1 (sif)	+++	++	+
RAC1 (Rac1)	++	+	+
RAC3 (Rac2)	+++	+++	++
WASHC4 (SWIP)	++	++	++
FLNA (cher)	++	+++	+++

Supplemental Figure 12. Actin regulatory genes interact with *PFN1* (*chic*), *EAAT2* (Eaat2) and *SOD1* *(sod1)* to induce bang sensitivity. (**A**) Genetic interaction assays of *SOD1* (*sod1*) and actin regulatory genes. Trans-heterozygous mutants (*Gene*^{LoF}/+*; sod1*^{LoF}/+) were assessed for bang sensitivity. Four epilepsy-associated actin regulatory genes (*sif, Rac2*, *SWIP* and *cher*) synergize with the *sod1*$^{T2A-GAL4}$ LoF allele to cause bang sensitivity (n = 26-61). (**B**) Summary of genetic interactions for bang sensitivity. Trans-heterozygous mutants (*GeneA*^{LoF}/+*; GeneB*^{LoF}/+) were generated and assessed for bang sensitivity. Average recovery time: +, 3-5 seconds; ++, 5-10 seconds; +++, 10-15 seconds. Flies were tested at 3-5 dpe. Data are presented as mean + SEM.

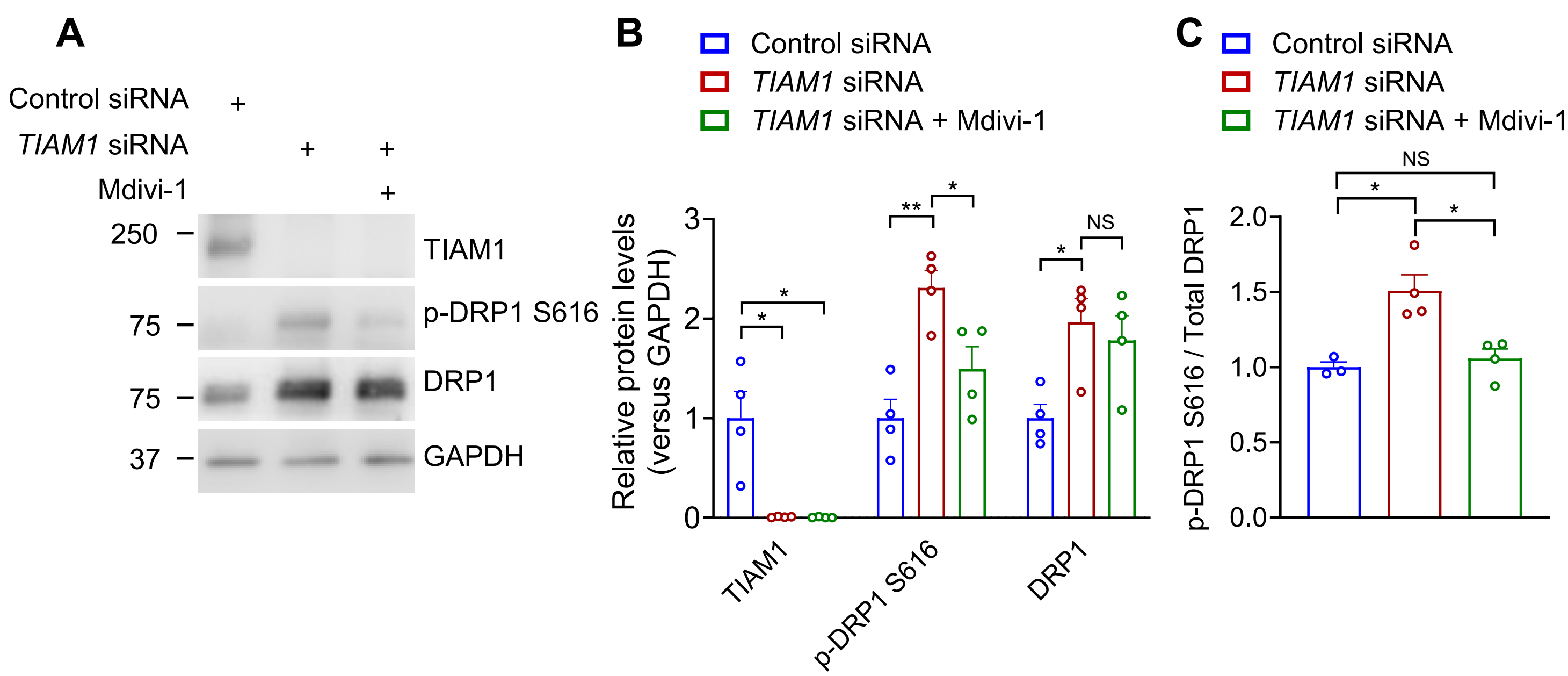

Supplemental Figure 13. Mdivi-1 reduces the p-DRP1 S616 levels in Daoy cells. (**A-C**) Western blotting (A) and quantification (B-C) of Daoy cells transfected with control siRNA or *TIAM1* siRNA for 72 hours, and treated with or without Mdivi-1 (20 μM, 24 hours) (n = 3-4). Data are presented as mean + SEM.

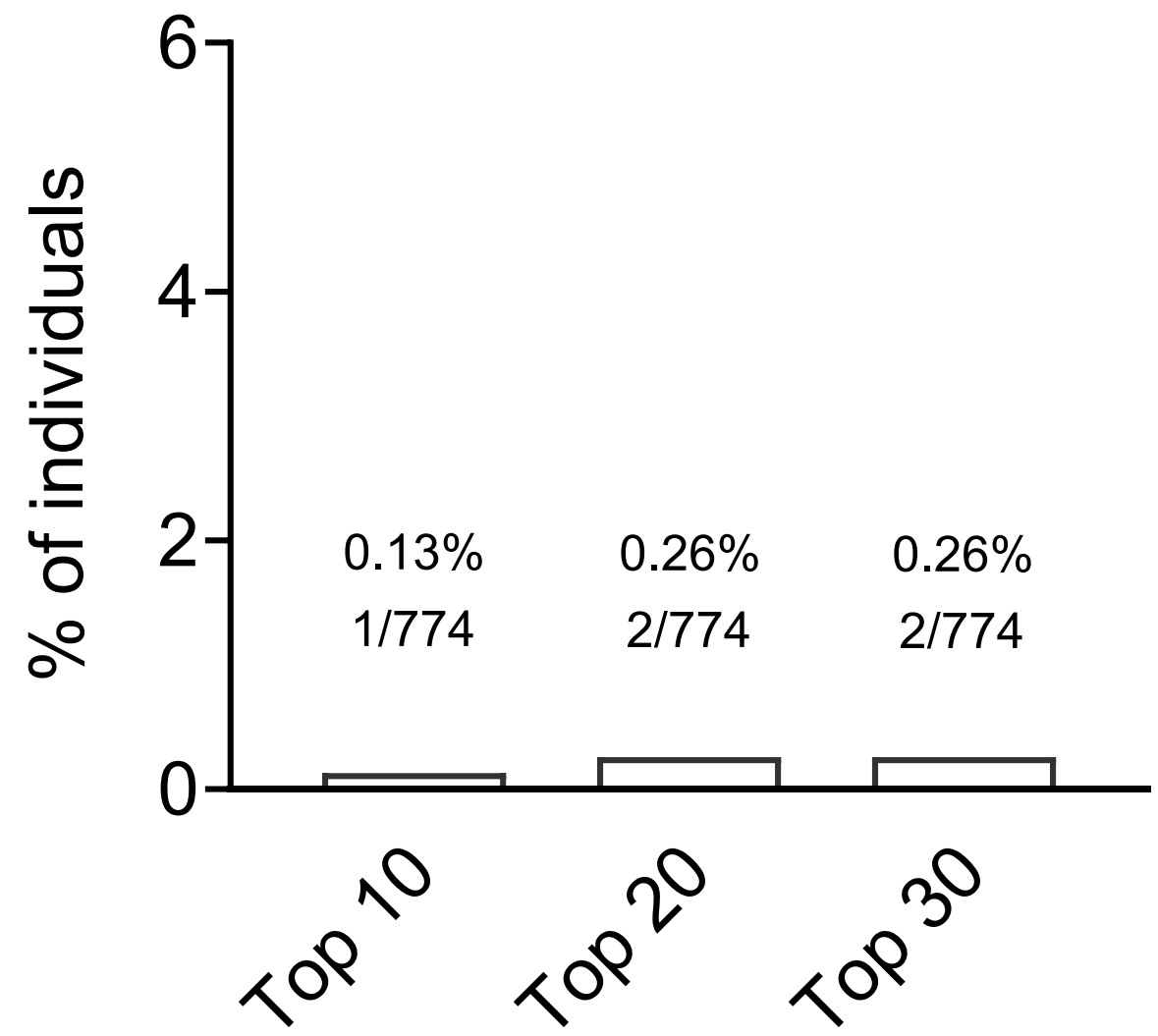

Supplemental Figure 14. The percentage of control individuals with two or more LoF variants in the AMG pathway genes is extremely low. Sequencing data of 774 individuals, randomly selected from 1000 Genomes Project dataset, were used as non-patient controls. For each individual, the top 10, 20, and 30 variants ranked by AI-MARRVEL (v1.1.3) were analyzed. The percentage of individuals with two or more LoF variants in the AMG gene set was calculated.

A A combination of LoF alleles identified from undiagnosed individuals with seizures

Individual	Gene	pLI	Variant Consequence	AIM	Zygosity	Gene Function	Gene-associated Phenotype / Inheritance
6	*AHI1*	0	frameshift	top 1	heterozygous	actin polymerization	Joubert syndrome (with seizures) / AR
	COQ8A	0	stop-gain	top 4	heterozygous	ROS inhibition	Coenzyme Q10 deficiency (with seizures) / AR
7	*VPS13A*	0	frameshift	top 4	heterozygous	actin polymerization	Choreoacanthocytosis (with seizures) / AR
	PCK2	0	frameshift	top 3	heterozygous	ROS inhibition	Mitochondrial PEPCK deficiency / AR

B Human genes, fly orthologs and alleles

Individual	Human gene	Fly ortholog	DIOPT score	Protein similarity	Protein identity	Fly LoF allele
6	*AHI1*	*wds*	2	47%	25%	*wds*^{7}
	COQ8A	*Coq8*	12	68%	50%	*Coq8*^{A}
7	*VPS13A*	*Vps13*	16	49%	29%	*Vps13*$^{T2A\text{-}GAL4}$
	PCK2	*Pepck2*	16	76%	65%	*Pepck2*^{e03788}

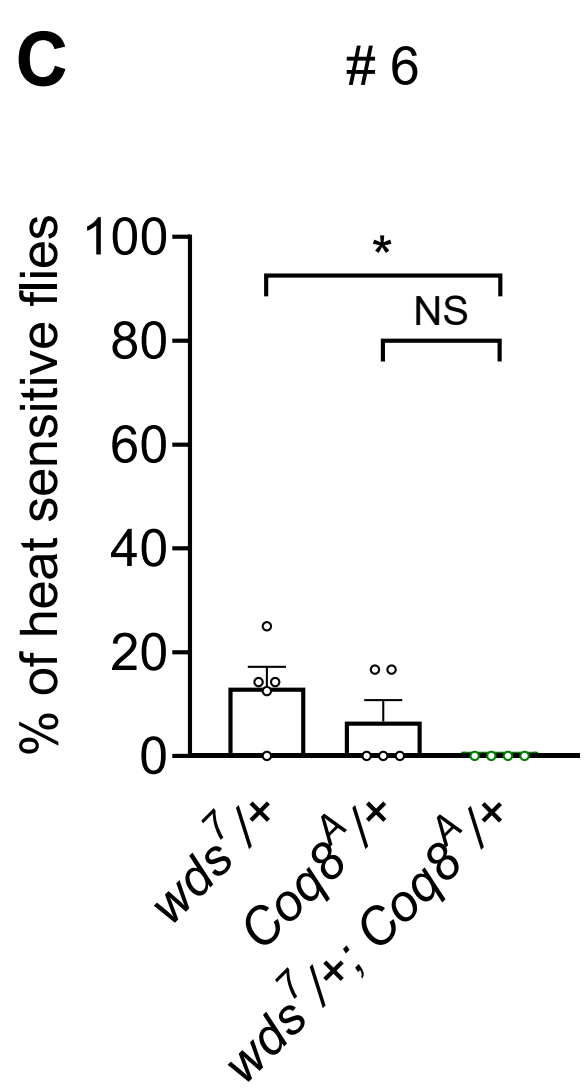

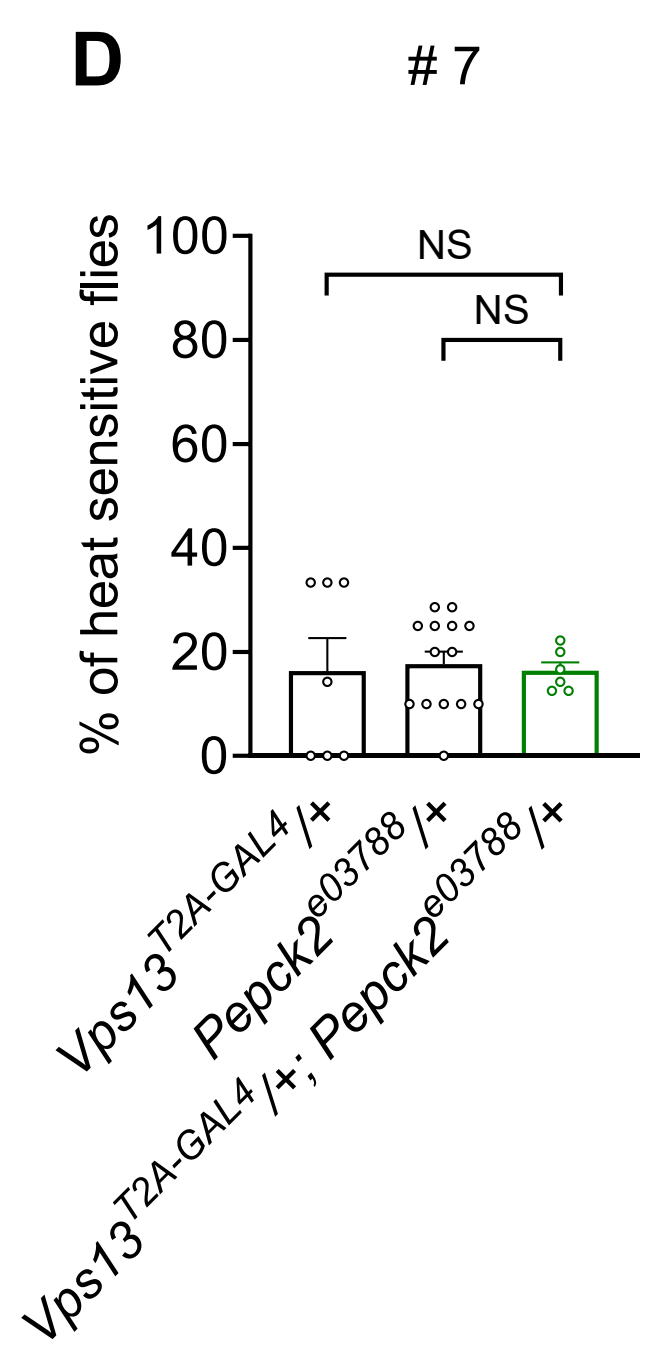

Supplemental Figure 15. Undiagnosed individuals with epilepsy who carry two pathogenic variants that do not exhibit synergistic or additive effects when tested in flies. (**A**) Table showing a combination of LoF alleles that affect actin polymerization and/or ROS production identified in undiagnosed individuals with seizures. References and clinical information can be found in Supplemental Table 3. AIM, AI-MARRVEL ranking. AR, autosomal recessive. (**B**) Human and fly orthologs and alleles used to generate trans-heterozygous mutants. (**C-D**) Heat sensitivity of adult flies. The allele combinations do not show synergistic or additive effects in flies (n = 4-14). Flies were tested at 6-9 dpe. Heat-sensitive flies are counted after heating at 42 °C for 50 seconds. Data are presented as mean + SEM.

A Penetrance of spontaneous seizure-like phenotypes (video recording at 25 °C)

Genotype	Total flies recorded	Flies with spontaneous seizure-like events
VGlut>sif RNAi	18	3
$sif^{T2A\text{-}GAL4}$/+; $chic^{01320}$/+	20	0
$Rac2^{KG05681b}$/+; $chic^{01320}$/+	20	7
$cher^{MI07480}$/+;$Eaat2^{CRIM\text{-}TG4}$/+	16	4
$cher^{MI07480}$/+; $sod1^{T2A\text{-}GAL4}$/+	20	0
$SdhA^{T2A\text{-}GAL4}$/+; $CG3803^{f04773}$/+	20	1
$SdhA^{T2A\text{-}GAL4}$/+; cdi^{MiMIC}/+	20	0
wds^{7}/+; $bt^{MB03788}$/+	20	1
$SdhA^{T2A\text{-}GAL4}$/+; $shot^{3}$/+	14	1
$CPT2^{T2A\text{-}GAL4}$/+; $a\text{-}Cat^{MiMIC}$/+	24	9

B Video recording at 29 °C

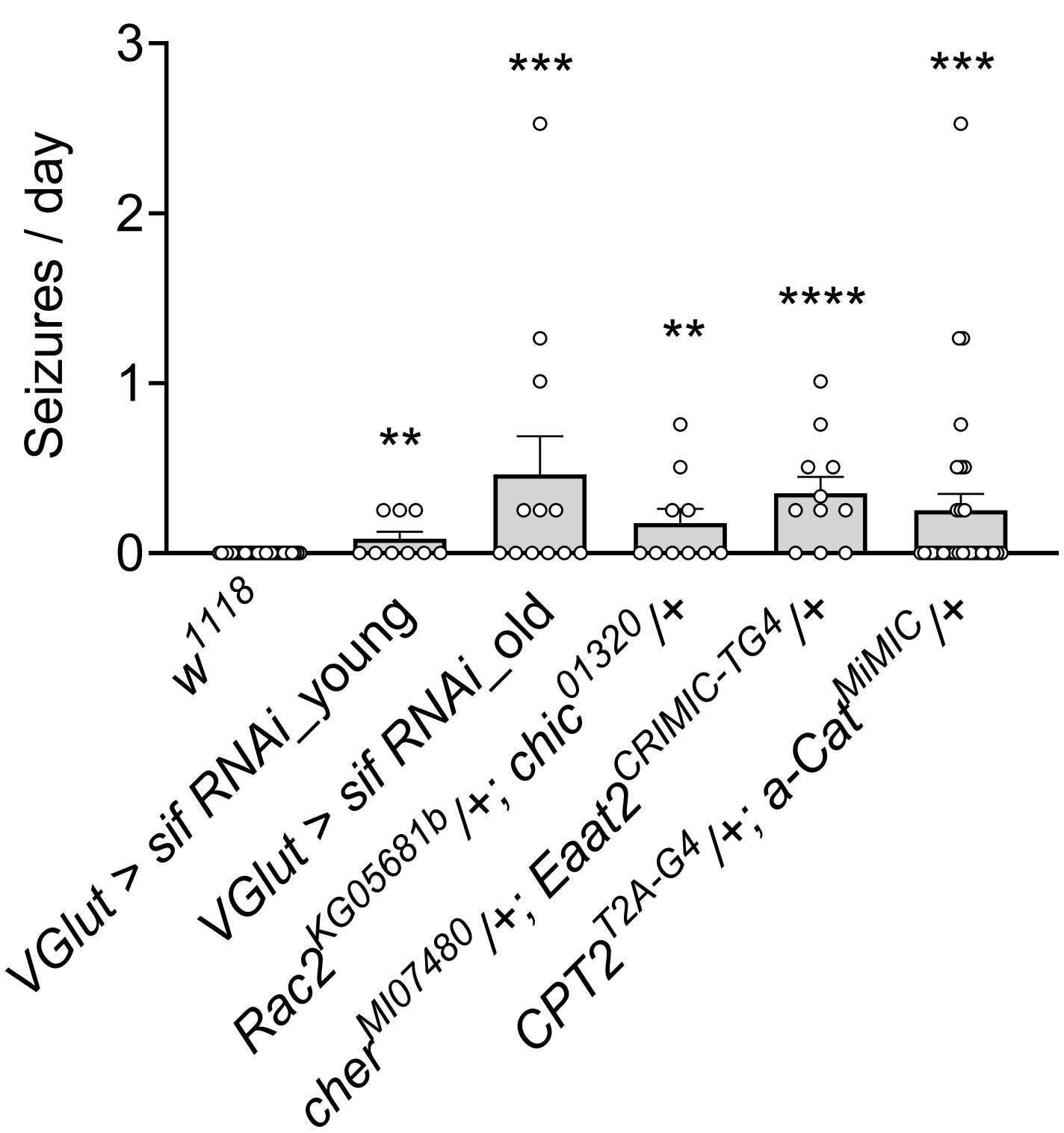

Supplemental Figure 16. Spontaneous seizure events of flies detected by the video-tracking system. Flies were placed into 48-well plates containing transparent food for video monitoring. The positions of each fly were recorded for four consecutive days, and an algorithm was used to identify seizure events. **(A)** The spontaneous seizure-like phenotypes of each group at 25 °C are presented in the table. **(B)** The spontaneous seizures detected at 29 °C were plotted. Each data point represents a single fly (n = 9-32). Flies were tested at 10-15 dpe. Old flies (*VGlut > sif RNAi*) were tested at ~30 dpe. The Wilcoxon Rank Sum Test was used to compare each group with the control (w^{1118}) group. Data are presented as mean + SEM.
