# Supplementary material for: Epilepsy-associated digenic variants affecting an actin/mitochondria/glutamate pathway promote seizure susceptibility
Source: J Clin Invest. 2026 Jul 16;136(17):e198696. doi: 10.1172/JCI198696 (PMC13528942; doi:10.1172/JCI198696)

Full unedited blot/gel for Figure 5B

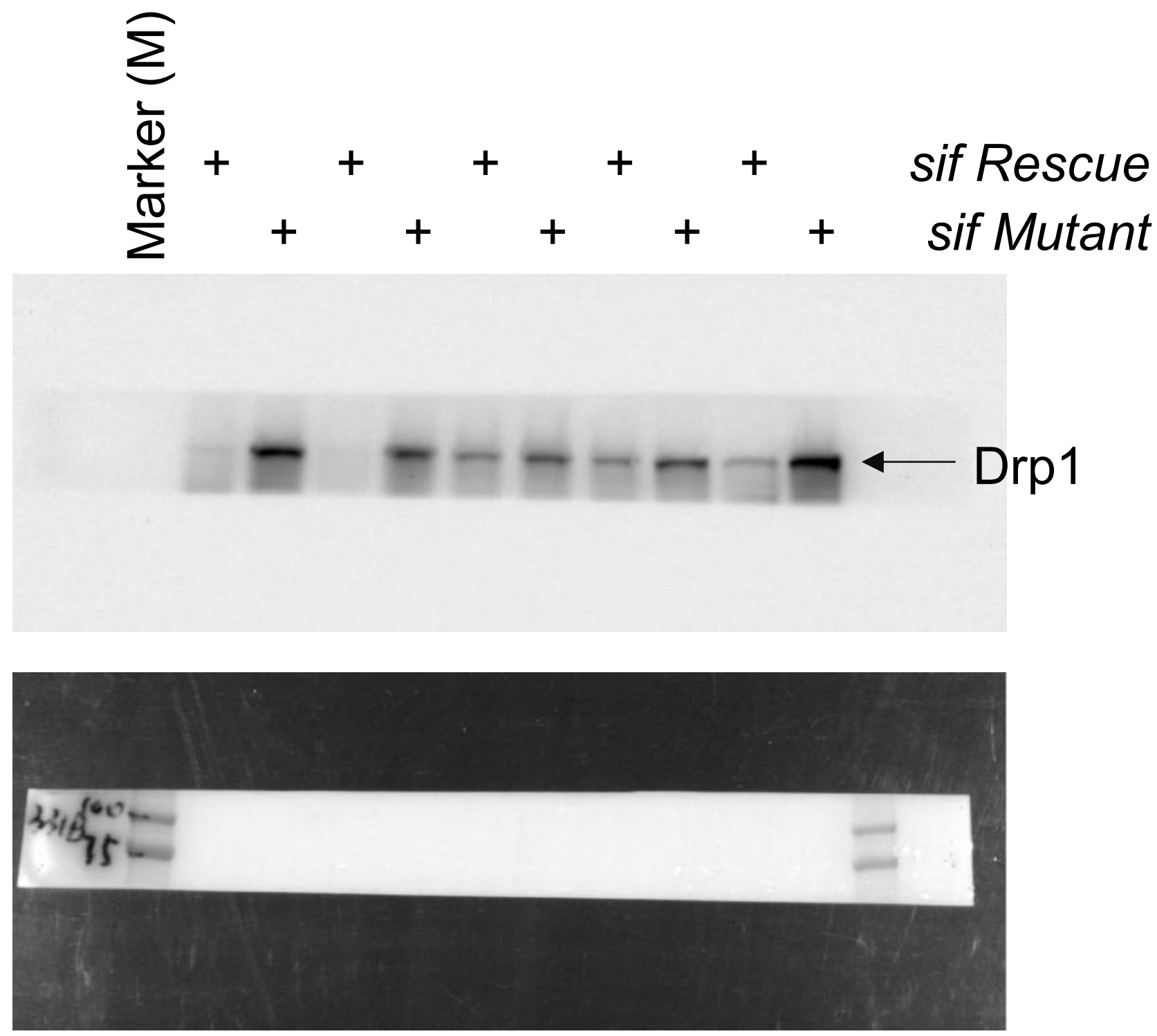

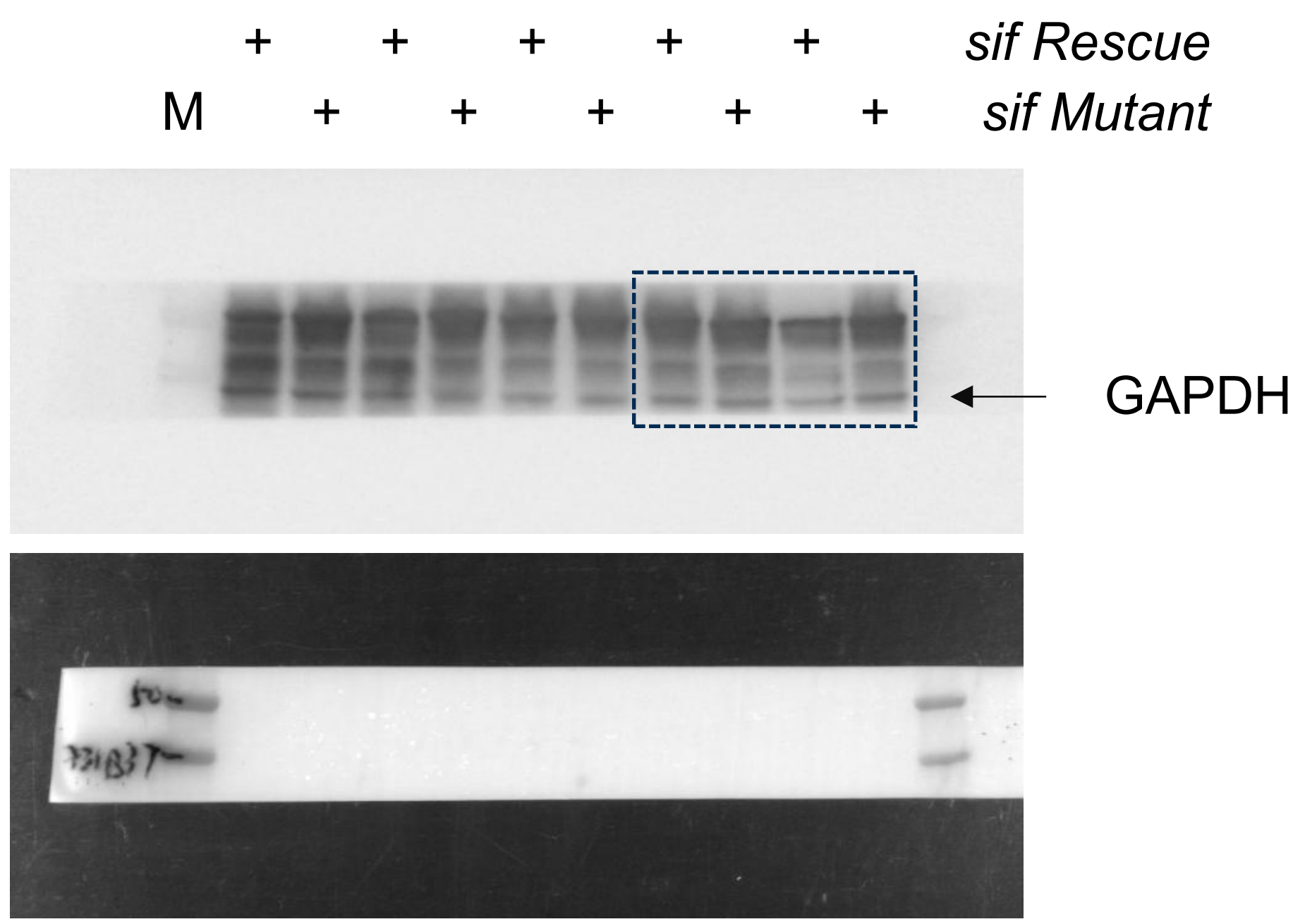

Full unedited blot/gel for Figure 5B

+ + + + + *sif Rescue*

M + + + + + *sif Mutant*

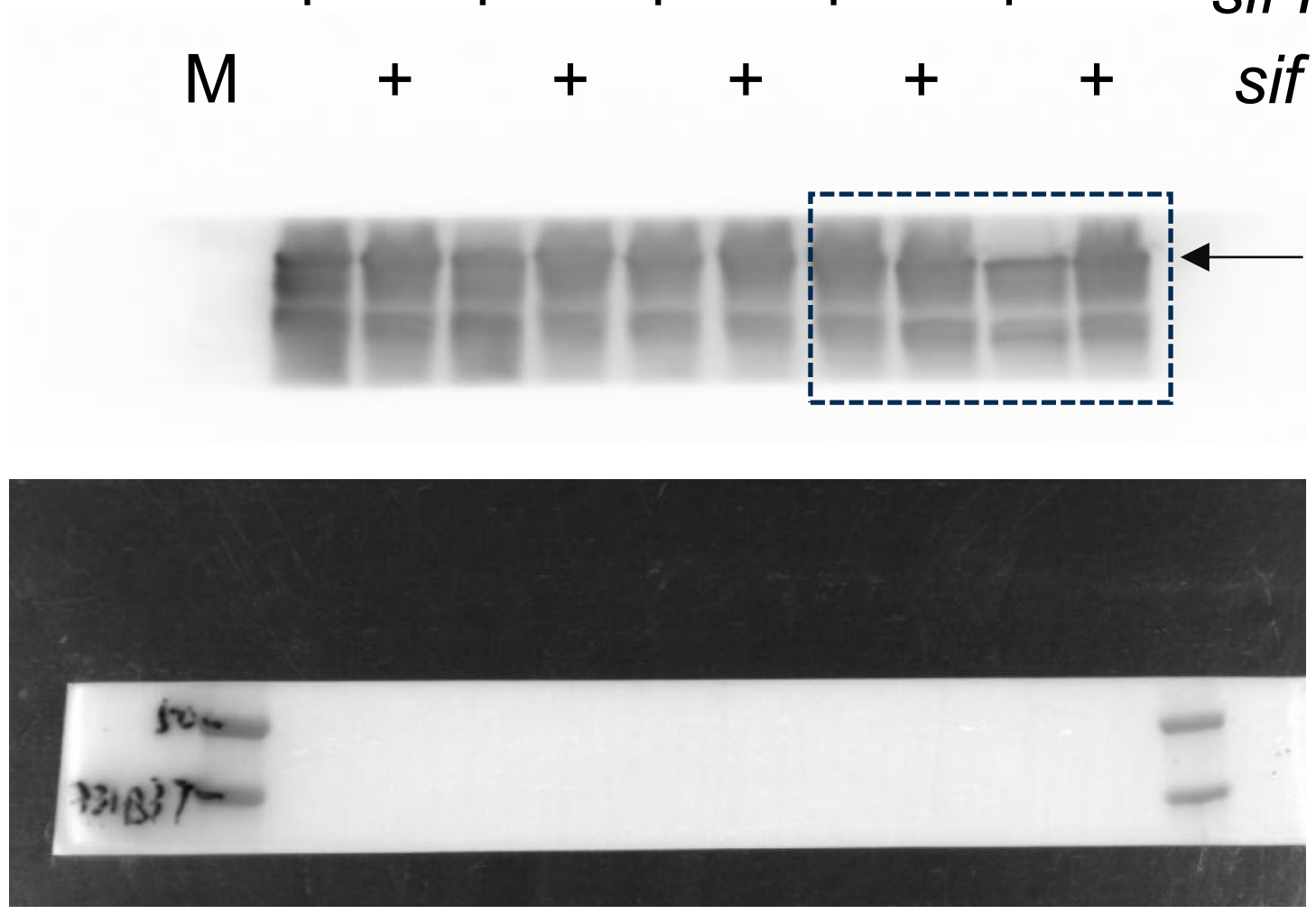

ATP5A

+ + + + + *sif Rescue*

M + + + + + *sif Mutant*

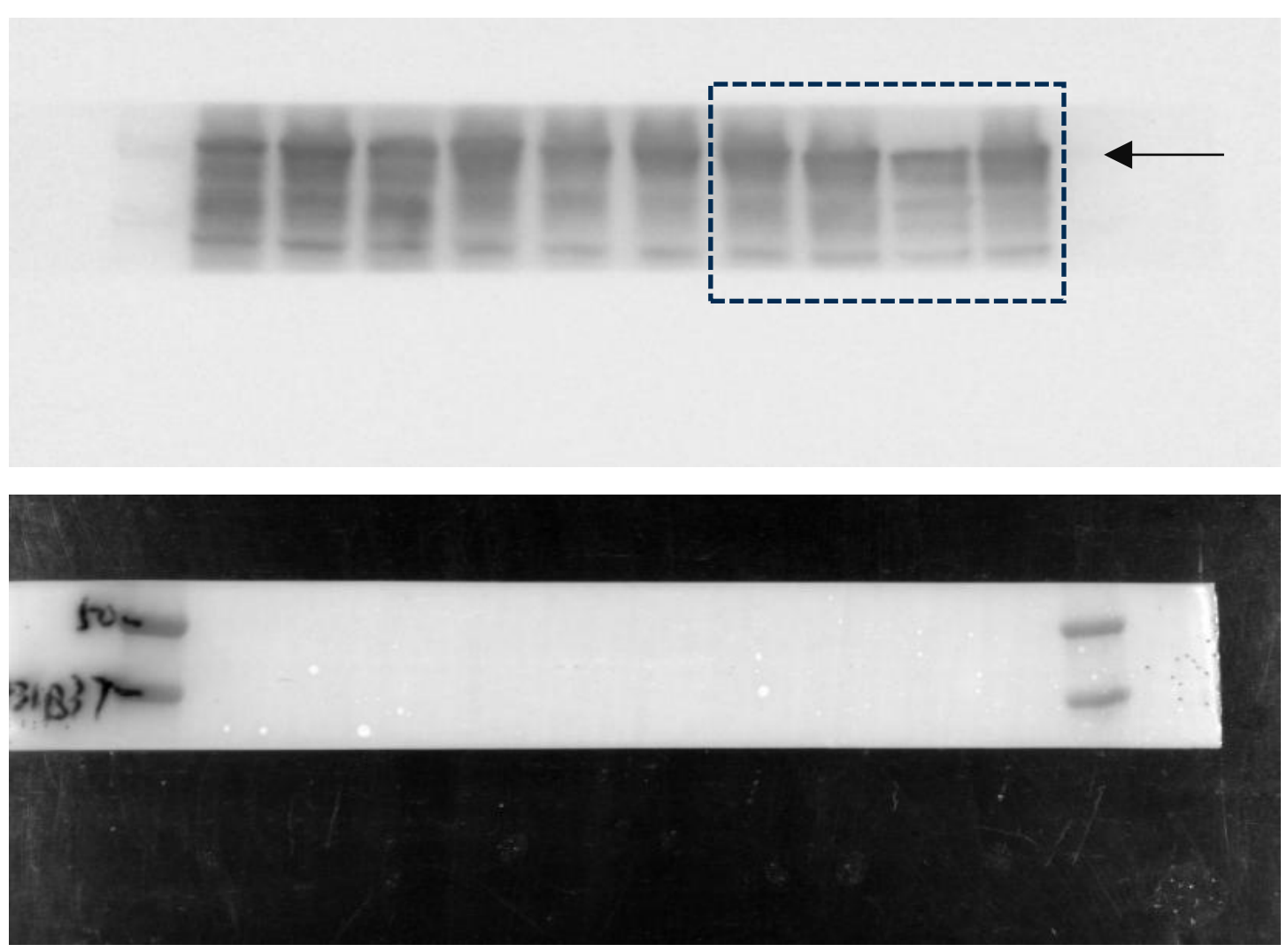

α-Tubulin

Full unedited blot/gel for Figure 7G

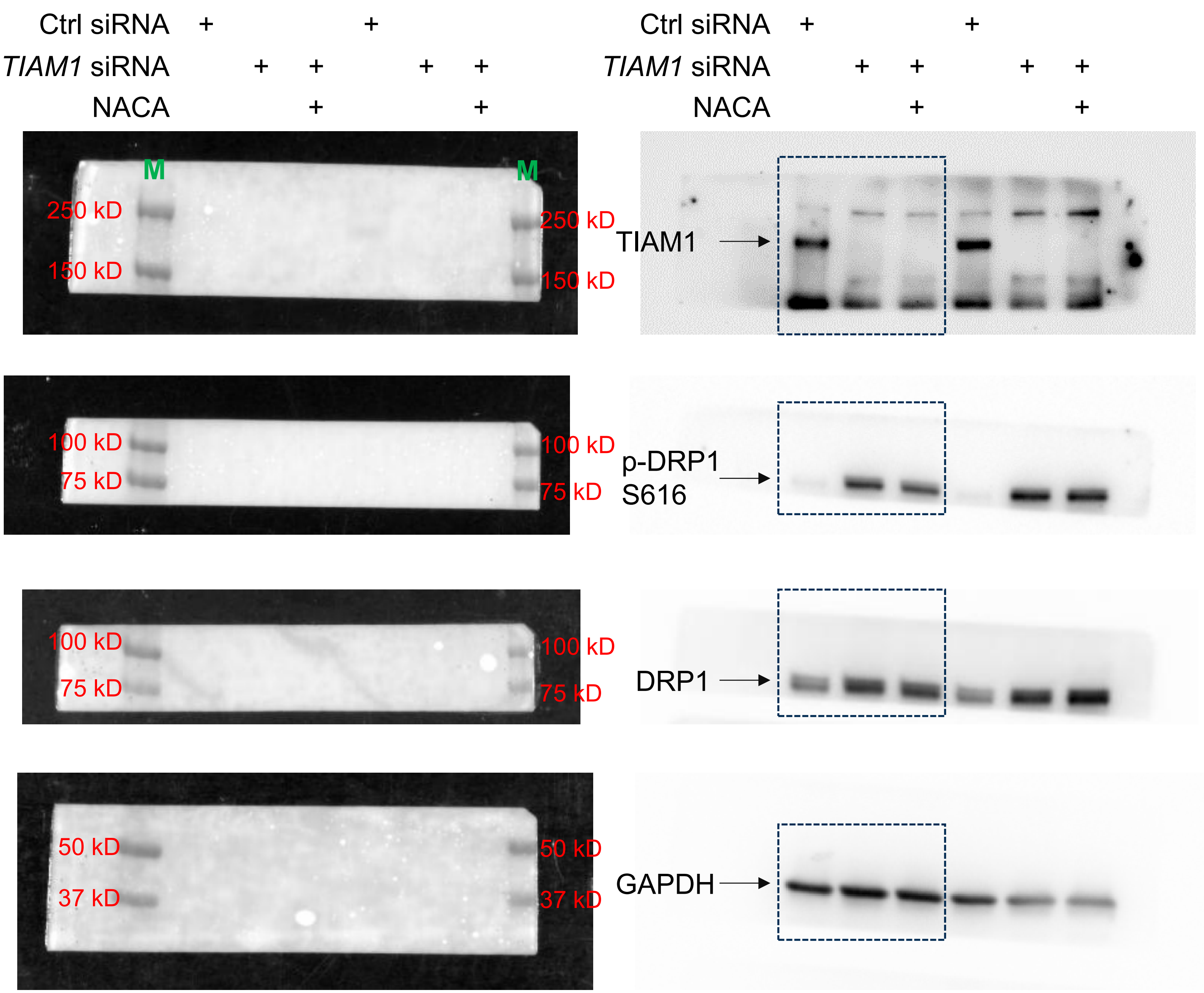

Full unedited blot/gel for Supplemental Figure 13A

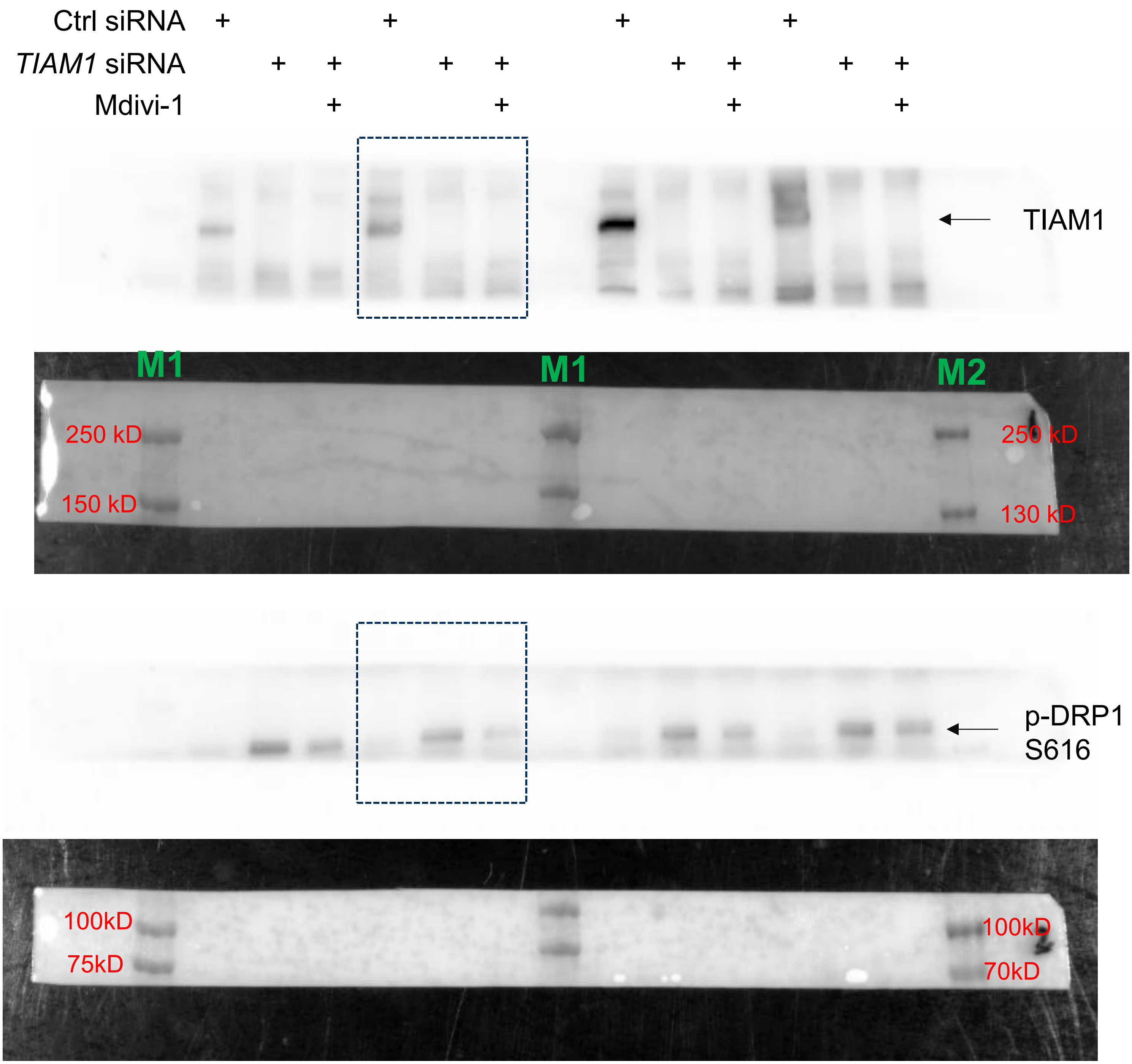

Full unedited blot/gel for Supplemental Figure 13A

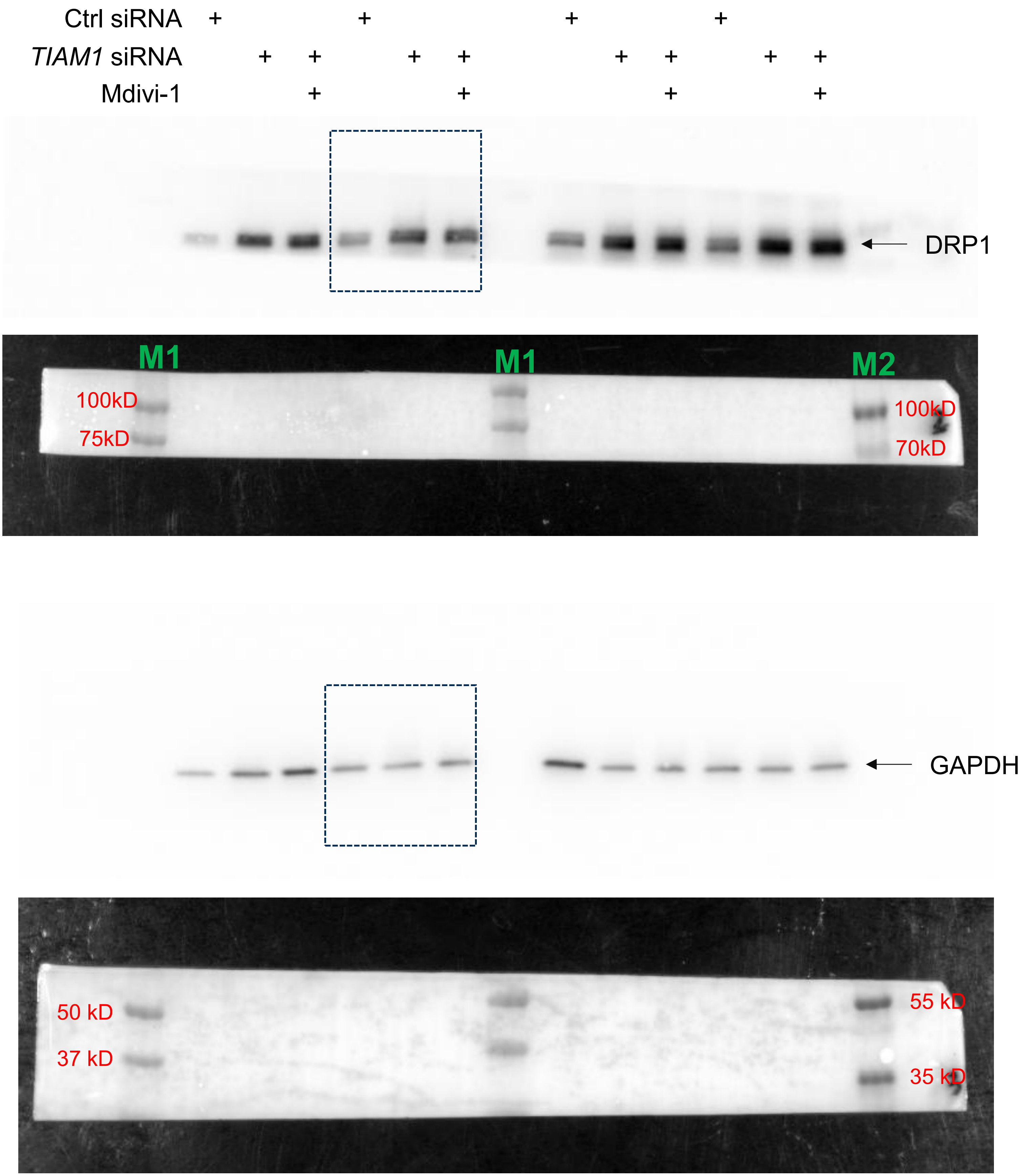

Supplement: Supplementary file 2 [file jci-136-198696-s045.pdf]
